# The new COST Action European Venom Network (EUVEN)—synergy and future perspectives of modern venomics

**DOI:** 10.1093/gigascience/giab019

**Published:** 2021-03-25

**Authors:** Maria Vittoria Modica, Rafi Ahmad, Stuart Ainsworth, Gregor Anderluh, Agostinho Antunes, Dimitris Beis, Figen Caliskan, Mauro Dalla Serra, Sebastien Dutertre, Yehu Moran, Ayse Nalbantsoy, Naoual Oukkache, Stano Pekar, Maido Remm, Bjoern Marcus von Reumont, Yiannis Sarigiannis, Andrea Tarallo, Jan Tytgat, Eivind Andreas Baste Undheim, Yuri Utkin, Aida Verdes, Aude Violette, Giulia Zancolli

**Affiliations:** Department of Biology and Evolution of Marine Organisms, Stazione Zoologica Anton Dohrn, Villa Comunale, 80121 Naples, Italy; Department of Biotechnology, Inland Norway University of Applied Sciences, Holsetgata 22, 2318 Hamar, Norway; Department of Tropical Disease Biology, Liverpool School of Tropical Medicine, L3 5QA, Liverpool, UK; National Institute of Chemistry, Hajdrihova 19, SI-1000 Ljubljana, Slovenia; CIIMAR/CIMAR, Interdisciplinary Centre of Marine and Environmental Research, University of Porto, Av. General Norton de Matos, 4450–208 Porto, Portugal; Department of Biology, Faculty of Sciences, University of Porto, Rua do Campo Alegre, 4169-007 Porto, Portugal; Biomedical Research Foundation, Academy of Athens, 4 Soranou Ephessiou St., 115 27 Athens, Greece; Eskisehir Osmangazi University, Faculty of Science and Letters, Department of Biology, TR-26040 Eskisehir, Turkey; Istituto di Biofisica, Consiglio Nazionale delle Ricerche, Via De Marini 6 - Torre di Francia, 16149 Genova, Italy; IBMM, Université de Montpellier, CNRS, ENSCM, Place Eugene Bataillon, 34095 Montpellier, France; Department of Ecology, Evolution and Behavior, Alexander Silberman Institute of Life Sciences, The Hebrew University of Jerusalem, Edmond J. Safra Campus - Givat Ram 9190401 Jerusalem, Israel; Ege University, Bioengineering Department, 180 Bornova, 35040 Izmir, Turkey; Institut Pasteur of Morocco, 1 Place Louis Pasteur, 20100 Casablanca, Morocco; Department of Botany and Zoology, Faculty of Science, Masaryk University, Kotlarska 2, 61137 Brno, Czechia; Department of Bioinformatics, University of Tartu, IMCB, Riia 23, 51010, Tartu, Estonia; Department of Insect Biotechnology, Justus Liebig University, Winchester Str. 2, 35394 Giessen, Germany; LOEWE Center for Translational Biodiversity Genomics, Senckenberganlage 25 D-60325 Frankfurt/Main, Germany; Department of Life and Health Sciences, University of Nicosia, 46 Makedonitissas Avenue, CY-2417 Nicosia, Cyprus; Department of Research infrastructures for Marine Biological Resources, Stazione Zoologica Anton Dohrn, Villa Comunale, 80121 Naples, Italy; Department of of Pharmaceutical and Pharmacological Sciences, KU Leuven, Herestraat 49, 3000 Leuven, Belgium; Centre for Ecological and Evolutionary Synthesis,Department of Biosciences, University of Oslo, 1066 Blindern, 0316 Oslo, Norway; Laboratory of Molecular Toxinology, Shemyakin-Ovchinnikov Institute of Bioorganic Chemistry, Russian Academy of Sciences, Miklukho-Maklaya, 16/10, 117997 Moscow, Russian Federation; Department of Biodiversity and Evolutionary Biology, Museo Nacional de Ciencias Naturales, Consejo Superior de Investigaciones Científicas, Calle de José Gutiérrez Abascal 2, 28006 Madrid, Spain; Department of Life Science, Natural History Museum, Cromwell Rd, South Kensington, London SW7 5BD, UK; Alphabiotoxine Laboratory, B-7911 Montroeul-au-Bois, Belgium; Department of Ecology and Evolution, University of Lausanne, UNIL Sorge Le Biophore, CH - 1015 Lausanne, Switzerland

**Keywords:** COST, venom, toxins, networking, interdisciplinarity

## Abstract

Venom research is a highly multidisciplinary field that involves multiple subfields of biology, informatics, pharmacology, medicine, and other areas. These different research facets are often technologically challenging and pursued by different teams lacking connection with each other. This lack of coordination hampers the full development of venom investigation and applications. The COST Action CA19144–European Venom Network was recently launched to promote synergistic interactions among different stakeholders and foster venom research at the European level.

## Background

Venomous species represent ∼15% of the global estimated animal biodiversity, are omnipresent in aquatic and terrestrial habitats, and evolved independently in all metazoan lineages in >100 instances [1]. Venoms are complex mixtures of bioactive compounds, mostly peptides and proteins, that evolved through millions of years of natural selection predominantly for predation and defense. Venom toxins are adaptive and highly convergent traits, extremely useful to understand the evolutionary mechanisms that link genotype, phenotype, and protein function.

In addition, toxins are streamlined to act fast at very low concentrations, being highly specific with key physiological targets of prey and/or predators (ion channels, enzymes, and cellular membrane components) [2]. Many toxins target the neuromuscular system, while others possess anticoagulant, cytolytic, anesthetic, and hypotensive activities [3]. These characteristics make them ideal candidates for biotechnological applications. To date, 10 animal-derived drugs have been approved and several others are in various stages of clinical trials to treat a wide array of diseases including cancer, hypertension, acute coronary syndromes, and chronic pain [4]. Besides medicine, venom toxins have great potential in other biotechnological fields: spider toxins to develop eco-friendly insecticides and other agrochemicals [4], ion channel blockers from cone snails and bees for cosmeceutical applications [5], and pore-forming toxins for sequencing and sensing technologies [6].

### Venomics as a multidisciplinary playground

**Figure 1:**
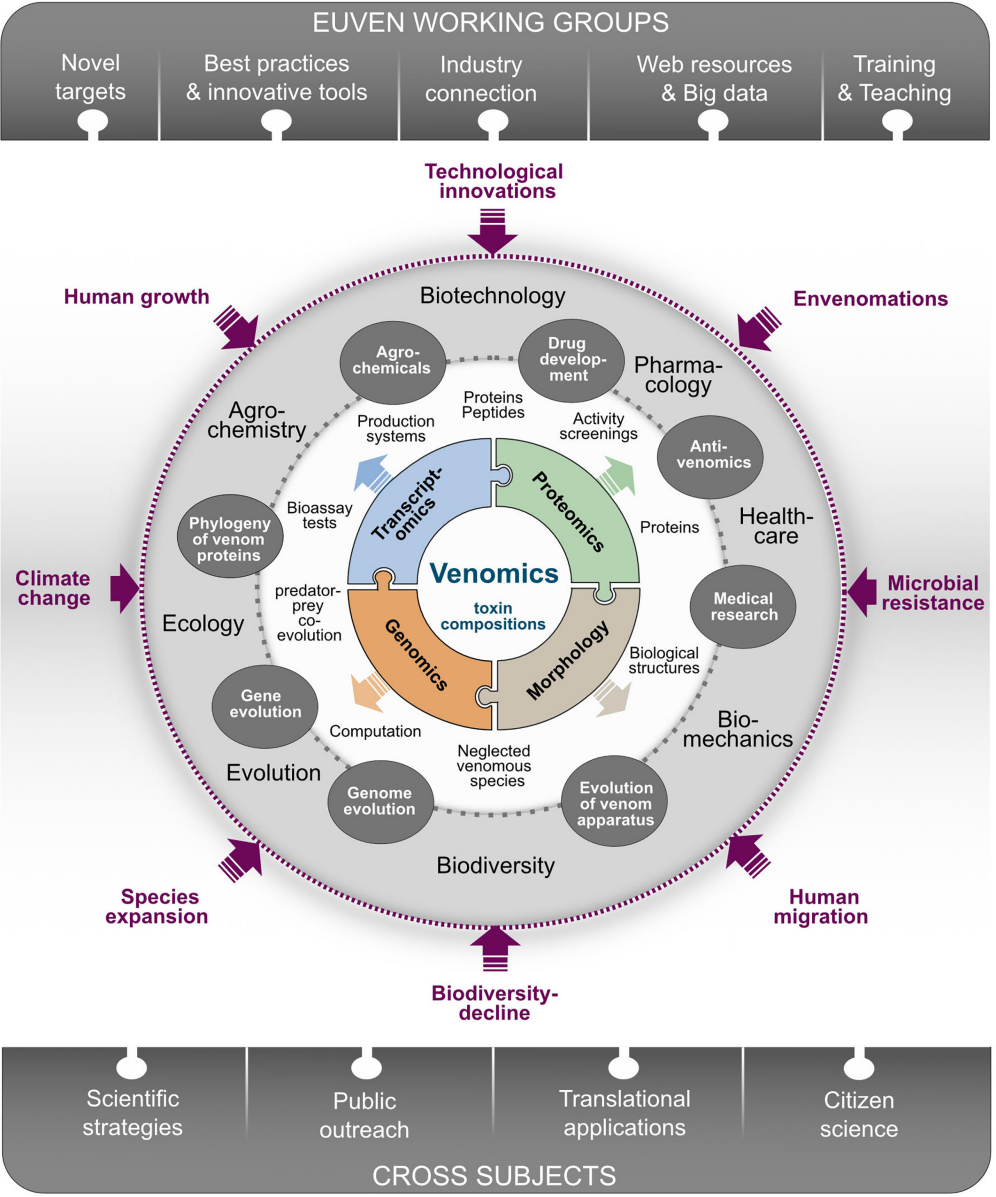
The multidisciplinary, integrative, and interconnected vision of venom research proposed in EUVEN. The centre is composed of modern morphology and -omics methods, in particular proteomics, transcriptomics, and genomics. In the surrounding white circle, main aspects of current venom research are indicated and summarized by the major topics in the dark grey ovals. These major topics are loosely associated with broader themes given in the light grey circle. The whole system is affected and interacts with outer drivers (purple). This integrative scheme is the heart of EUVEN, in which 5 major Working Groups focus on these topics and methods (top dark grey bar; see text for details). The cross subjects in which the scientific, technological, and socio-economic impact of EUVEN will be realized are outlined in the bottom dark grey bar.

Venom investigation involves many scientific disciplines that in recent years have undergone great technological improvements (Fig. 1). These fast-evolving technologies foster venomics research but also bring new challenges, necessitating considerable integrative expertise.

High-throughput techniques have facilitated the characterization of complex venoms even in non-model organisms [7]. Transcriptomic data obtained by the latest RNA-sequencing technologies are often integrated with bottom-up proteomics, in which high-performance liquid chromatography is coupled with tandem mass spectrometry. This proteo-transcriptomics approach allows the detection of low-copy transcripts and post-translational modifications, and a precise relative quantification of expressed proteins. Integration of genomic data is still uncommon, despite its promise for elucidating evolutionary and regulatory patterns of venom compounds.

Bioinformatics pipelines are used for similarity-based screening and identification of promising candidates. Afterwards, the peptide and protein toxins are synthesized via solid-phase peptide synthesis and regioselective folding, or by a variety of different recombinant expression systems to obtain a realistic folding pattern. When separation and isolation of each compound is not achievable owing to the low quantity of raw venom, these procedures can yield amounts of proteins suitable for subsequent activity testing, although they require extensive optimization for each component.

Activity screening mostly relies on electrophysiology that is applied on multiple neuroreceptors, ligand-gated and voltage-gated ion channels involved in neurodegenerative and drug dependency disorders, in immune system regulation, anesthesia, and neuropathic pain. Electrophysiology also includes *ex vivo* assays and bi-dimensional array assays on tissue preparations for neurological disorders and trauma. Other activity screening tests target hormonal pathways, cancer, cardiovascular or inflammatory disorders, diabetes and obesity, and infectious diseases. Bioactivity-driven identification of novel compounds is further tested by *in vivo* phenotypic screens [8].

In addition, biophysical approaches such as X-ray crystallography, nuclear magnetic resonance spectroscopy, surface plasmon resonance spectroscopy, isothermal titration calorimetry, and micro-computer tomography have become key components of drug discovery platforms and venom systems identification. *In silico* approaches, such as molecular modeling, have also become widely used for studying venom components, providing structural information and theoretical understanding of the molecular mechanisms of toxin action [9].

### The COST Action EUVEN: from fragmentation to integration

**Figure 2:**
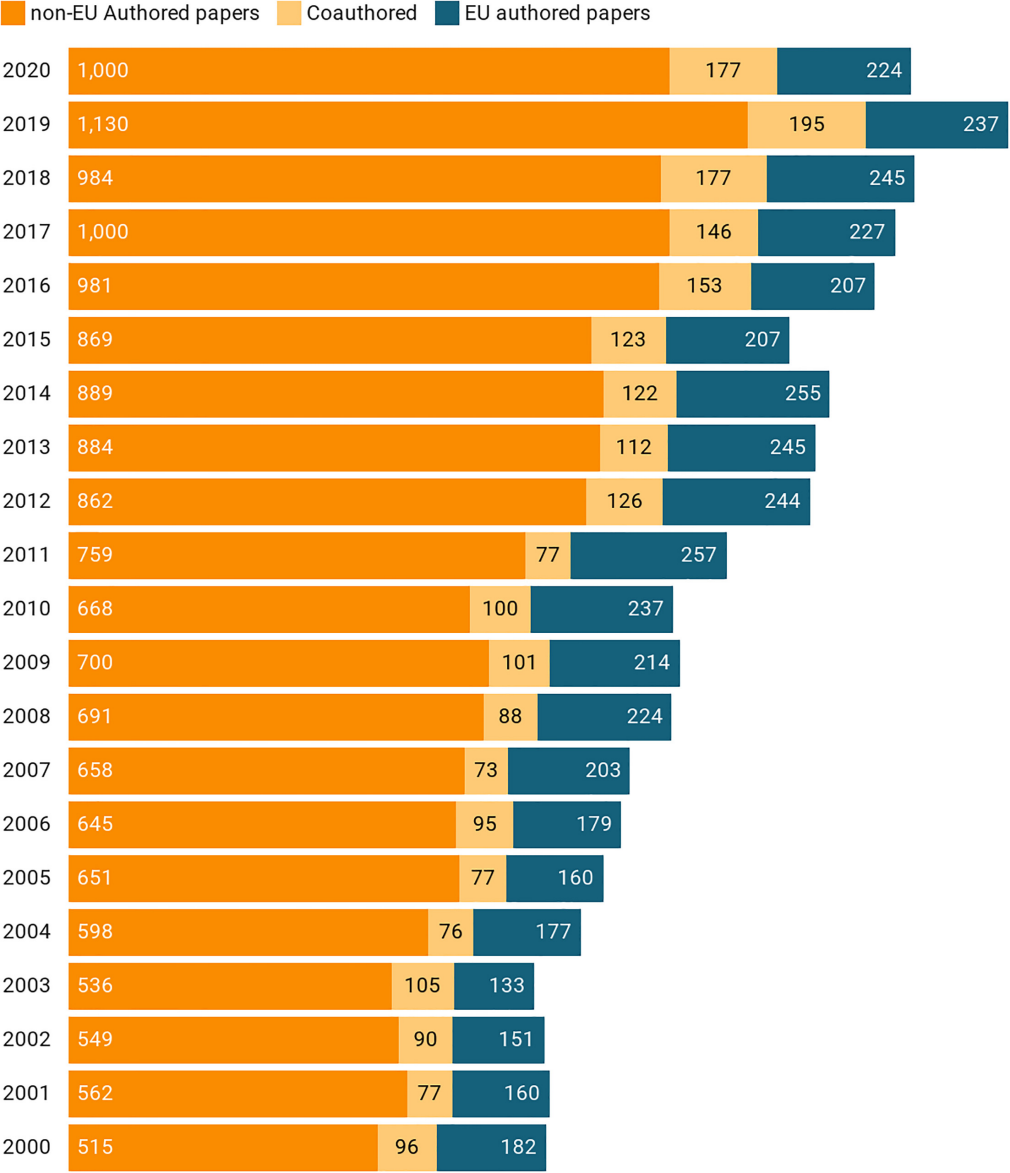
Number of indexed publications on venom between the years 2000 and 2020, comparing EU and extra-EU authors' contributions to venom research. The non-EU–authored publications doubled in the past 20 years. By contrast, the EU-only–authored ones only increased by ∼20%. The word “venom” wasted as query for “topic.” EU and non-EU countries were then excluded to obtain publication lists of extra-EU authors only and EU authors only, respectively (Source: Web of Science, last updated December 2020).

The different facets of venomics are typically pursued by different research groups, whose level of collaboration in the EU is not adequate to face the increasing challenges in venomics research, as reflected by the decreased relative contributions of EU scientists to global venom research in the past 20 years (Fig. 2).

The European Venom Network COST Action CA19144 (EUVEN) [10] was launched in October 2020 to promote an efficient exchange of ideas, knowledge, and benchmarks and to involve all relevant stakeholders to foster European venom research. The European Cooperation in Science and Technology (COST) Association has operated since 1971 funding bottom-up networks that run for 4 years to boost research, innovation, and careers through a series of collaborative activities including workshops, conferences, working group (WG) meetings, training schools, short-term scientific missions, dissemination, and outreach.

In bringing together experts from all relevant fields, EUVEN develops protocols on best practices in venomics and identifies the most promising novel technological tools, animal models, and untapped physiological targets to be integrated into current venom research. Stronger collaborations with non-academic stakeholders, especially with small and medium enterprises (SMEs), are enforced as a prerequisite for promoting biomedical, diagnostic, agrochemical, cosmeceutical, or nanobiotechnological applications of venom compounds. EUVEN also aims to involve non-professional societies to foster biodiversity-based research with the support of natural history museums. Our major research and capacity-building objectives will be tackled through the engagement of participants (currently from 31 countries) in 5 WGs.

WG1: Novel targets in venom research—In this WG, novel targets are established and validated to expand and diversify the current main focus of the research community on just a few model organisms, diseases, and molecular targets.

WG2: Best practices and innovative tools—The aim of this WG is to validate and develop best research practices to minimize variation and gather reproducible and comparable results in all experimental and analytical steps contributing to venom investigation. Additionally, the integration of innovative tools in venom research will be evaluated.

WG3: Interaction with industry—The focus of WG3 is the interplay between academic researchers and industrial partners to transform researchers' discoveries and knowledge into innovations for society. EUVEN supports different initiatives to encourage participation of SMEs in meetings and discussions and to increase the appeal of venom research for industries.

WG4: Web resources—Available web resources for venom research currently lack a streamlined synergism. WG4 aims at integrating databases in a single repository that will also implement the most useful computational analytical tools for the detection of relevant features, including folds/activities of potential applicative interest.

WG5: Training—Given the multidisciplinary nature of venom research, EUVEN plans to offer extensive training opportunities in all relevant venom research disciplines.

## Conclusion

The new COST Action EUVEN provides a flexible platform for scientists to overcome the lack of coordination, tools, and resources, through the development of a fully synergistic network. To guarantee the coverage of the diverse topics of interest in EUVEN and build an effective network across Europe and beyond, it is fundamental to engage the broadest participation possible from all COST participating countries. Near-neighbor and international partner countries can also request to join EUVEN and participate in networking activities.

We believe that building an effective network that is able to bridge different scientific disciplines and sectors constitutes a fundamental prerequisite to fully develop the extraordinarily transformative potential of venom research.

## Data Availability

Not applicable.

## Abbreviations

COST: European Cooperation in Science and Technology; EUVEN: European Venom Network COST Action CA19144; SME: small and medium enterprise; WG: working group.

## Competing Interests

The authors declare that they have no competing interests.

## Funding

The authors acknowledge support from the European Cooperation in Science and Technology (COST) through the Action CA19144 EUVEN. AV was supported by the European Union’s Horizon 2020 research and innovation program through a Marie Sklodowska-Curie Individual Fellowship (grant 841576). GZ was supported by the European Union’s Horizon 2020 research and innovation program through a Marie Sklodowska-Curie Individual Fellowship (grant 845674).

## Authors' Contributions

Conceptualization, M.V.M., A.A., G.A., S.D., B.M.v.R., and S.P.; writing—original draft preparation, M.V.M.; figures preparation, B.M.v.R., A.T.; writing—review and editing, all authors.; project administration, M.V.M, G.A., A.T. Except for the first, authors are listed alphabetically with respect to last name. All authors have read and agreed to the published version of the manuscript. Parts of this paper are derived from the Memorandum of Understanding for the implementation of the COST Action “European Venom Network” (EUVEN) CA19144.

## Supplementary Material

giab019_GIGA-D-21-00035_Original_Submission

giab019_GIGA-D-21-00035_Revision_1

giab019_Response_to_Reviewer_Comments_Original_Submission

giab019_Reviewer_1_Report_Original_Submission Juan Calvete -- 2/10/2021 Reviewed

giab019_Reviewer_2_Report_Original_Submission Andrew J Mason -- 2/16/2021 Reviewed
