# Corrigendum to: The new COST Action European Venom Network (EUVEN)—synergy and future perspectives of modern venomics

**DOI:** 10.1093/gigascience/giab102

**Published:** 2021-12-29

**Authors:** Maria Vittoria Modica, Rafi Ahmad, Stuart Ainsworth, Gregor Anderluh, Agostinho Antunes, Dimitris Beis, Figen Caliskan, Mauro Dalla Serra, Sebastien Dutertre, Yehu Moran, Ayse Nalbantsoy, Naoual Oukkache, Stano Pekar, Maido Remm, Bjoern Marcus von Reumont, Yiannis Sarigiannis, Andrea Tarallo, Jan Tytgat, Eivind Andreas Baste Undheim, Yuri Utkin, Aida Verdes, Aude Violette, Giulia Zancolli

**Affiliations:** Department of Biology and Evolution of Marine Organisms, Stazione Zoologica Anton Dohrn, Villa Comunale, 80121 Naples, Italy; Department of Biotechnology, Inland Norway University of Applied Sciences, Holsetgata 22, 2318 Hamar, Norway; Department of Tropical Disease Biology, Liverpool School of Tropical Medicine, L3 5QA, Liverpool, UK; National Institute of Chemistry, Hajdrihova 19, SI-1000 Ljubljana, Slovenia; CIIMAR/CIMAR, Interdisciplinary Centre of Marine and Environmental Research, University of Porto, Av. General Norton de Matos, 4450–208 Porto, Portugal; Department of Biology, Faculty of Sciences, University of Porto, Rua do Campo Alegre, 4169-007 Porto, Portugal; Biomedical Research Foundation, Academy of Athens, 4 Soranou Ephessiou St., 115 27 Athens, Greece; Eskisehir Osmangazi University, Faculty of Science and Letters, Department of Biology, TR-26040 Eskisehir, Turkey; Istituto di Biofisica, Consiglio Nazionale delle Ricerche, Via De Marini 6 - Torre di Francia, 16149 Genova, Italy; IBMM, Universite de Montpellier, CNRS, ´ ENSCM, Place Eugene Bataillon, 34095 Montpellier, France; Department of Ecology, Evolution and Behavior, Alexander Silberman Institute of Life Sciences, The Hebrew University of Jerusalem, Edmond J. Safra Campus - Givat Ram 9190401 Jerusalem, Israel; Ege University, Bioengineering Department, 180 Bornova, 35040 Izmir, Turkey; Institut Pasteur of Morocco, 1 Place Louis Pasteur, 20100 Casablanca, Morocco; Department of Botany and Zoology, Faculty of Science, Masaryk University, Kotlarska 2, 61137 Brno, Czechia; Department of Bioinformatics, University of Tartu, IMCB, Riia 23, 51010, Tartu, Estonia; Department of Insect Biotechnology, Justus Liebig University, Winchester Str. 2, 35394 Giessen, Germany; LOEWE Center for Translational Biodiversity Genomics, Senckenberganlage 25 D-60325 Frankfurt/Main, Germany; Department of Life and Health Sciences, University of Nicosia, 46 Makedonitissas Avenue, CY-2417 Nicosia, Cyprus; Department of Research infrastructures for Marine Biological Resources, Stazione Zoologica Anton Dohrn, Villa Comunale, 80121 Naples, Italy; Department of of Pharmaceutical and Pharmacological Sciences, KU Leuven, Herestraat 49, 3000 Leuven, Belgium; Centre for Ecological and Evolutionary Synthesis, Department of Biosciences, University of Oslo, 1066 Blindern, 0316 Oslo, Norway; Laboratory of Molecular Toxinology, Shemyakin-Ovchinnikov Institute of Bioorganic Chemistry, Russian Academy of Sciences, Miklukho-Maklaya, 16/10, 117997 Moscow, Russian Federation; Department of Biodiversity and Evolutionary Biology, Museo Nacional de Ciencias Naturales, Consejo Superior de Investigaciones Cient´ıficas, Calle de Jose Guti ´ errez ´ Abascal 2, 28006 Madrid, Spain; Department of Life Science, Natural History Museum, Cromwell Rd, South Kensington, London SW7 5BD, UK; Alphabiotoxine Laboratory, B-7911 Montroeul-au-Bois, Belgium; Department of Ecology and Evolution, University of Lausanne, UNIL Sorge Le Biophore, CH - 1015 Lausanne, Switzerland

## Correction

In the final publication of “The new COST Action European Venom Network (EUVEN)—synergy and future perspectives of modern venomics” some of the funding details were unfortunately missed. It should have been noted in the Funding section: “AV was supported by the European Union's Horizon 2020 research and innovation program through a Marie Sklodowska-Curie Individual Fellowship (grant 841 576). GZ was supported by the European Union's Horizon 2020 research and innovation program through a Marie Sklodowska-Curie Individual Fellowship (grant 845 674)". This has now been corrected online.

