# Supplementary material for: The new COST Action European Venom Network (EUVEN)—synergy and future perspectives of modern venomics
Source: Gigascience. 2021 Mar 25;10(3):giab019. doi: 10.1093/gigascience/giab019 (PMC7992391; doi:10.1093/gigascience/giab019)

GigaScience

The new COST Action European Venom Network (EUVEN) – synergy and future perspectives of modern venomics

--Manuscript Draft--

**Manuscript Number:**	GIGA-D-21-00035
**Full Title:**	The new COST Action European Venom Network (EUVEN) – synergy and future perspectives of modern venomics
**Article Type:**	Commentary
**Funding Information:**	European Cooperation in Science and Technology (COST) (CA19144) — Not applicable
**Abstract:**	Venom research is a highly multidisciplinary field that involves multiple subfields of biology, informatics, pharmacology, medicine and other areas. These different research facets are often technologically challenging and pursued by different teams lacking connection with each other. This lack of coordination hampers the full development of venom investigation and applications. The COST Action CA19144 – European Venom Network, was recently launched to promote synergistic interactions among different stakeholders and foster venom research at the European level.
**Corresponding Author:**	Maria Vittoria Modica, Ph.D.<br>Stazione Zoologica Anton Dohrn<br>Napoli, ITALY
**Corresponding Author Secondary Information:**	
**Corresponding Author's Institution:**	Stazione Zoologica Anton Dohrn
**Corresponding Author's Secondary Institution:**	
**First Author:**	Maria Vittoria Modica
**First Author Secondary Information:**	
**Order of Authors:**	Maria Vittoria Modica
	Rafi Ahmad
	Stuart Ainsworth
	Gregor Anderluh
	Agostinho Antunes
	Dimitri Beis
	Figen Caliskan
	Mauro Dalla Serra
	Sebastien Dutertre
	Yehu Moran
	Ayse Nalbantsoy
	Naoual Oukkache
	Stano Pekar
	Maido Remm
	Bjoern Marcus von Reumont
	Yiannis Sarigiannis
	Andrea Tarallo

	Jan Tytgat
	Yuri Utkin
	Aida Verdes
	Aude Violette
	Giulia Zancolli
Order of Authors Secondary Information:	
Additional Information:	
Question	Response
Are you submitting this manuscript to a special series or article collection?	No
**Experimental design and statistics**<br><br>Full details of the experimental design and statistical methods used should be given in the Methods section, as detailed in our Minimum Standards Reporting Checklist. Information essential to interpreting the data presented should be made available in the figure legends.<br><br>Have you included all the information requested in your manuscript?	No
If not, please give reasons for any omissions below.<br><br>as follow-up to "**Experimental design and statistics**<br><br>Full details of the experimental design and statistical methods used should be given in the Methods section, as detailed in our Minimum Standards Reporting Checklist. Information essential to interpreting the data presented should be made available in the figure legends.<br><br>Have you included all the information requested in your manuscript?<br><br>"	Not applicable
**Resources**	No

A description of all resources used, including antibodies, cell lines, animals and software tools, with enough information to allow them to be uniquely identified, should be included in the Methods section. Authors are strongly encouraged to cite Research Resource Identifiers (RRIDs) for antibodies, model organisms and tools, where possible.<br><br>Have you included the information requested as detailed in our Minimum Standards Reporting Checklist?	
If not, please give reasons for any omissions below.<br><br>as follow-up to "**Resources**<br><br>A description of all resources used, including antibodies, cell lines, animals and software tools, with enough information to allow them to be uniquely identified, should be included in the Methods section. Authors are strongly encouraged to cite Research Resource Identifiers (RRIDs) for antibodies, model organisms and tools, where possible.<br><br>Have you included the information requested as detailed in our Minimum Standards Reporting Checklist?<br><br>"	Not appplicable
**Availability of data and materials**<br><br>All datasets and code on which the conclusions of the paper rely must be either included in your submission or deposited in publicly available repositories (where available and ethically appropriate), referencing such data using a unique identifier in the references and in the "Availability of Data and Materials" section of your manuscript.	No

Powered by Editorial Manager® and ProduXion Manager® from Aries Systems Corporation

Have you have met the above requirement as detailed in our Minimum Standards Reporting Checklist?	
If not, please give reasons for any omissions below.<br><br>as follow-up to "**Availability of data and materials**<br><br>All datasets and code on which the conclusions of the paper rely must be either included in your submission or deposited in publicly available repositories (where available and ethically appropriate), referencing such data using a unique identifier in the references and in the “Availability of Data and Materials” section of your manuscript.<br><br>Have you have met the above requirement as detailed in our Minimum Standards Reporting Checklist?<br><br>"	Not applicable

Powered by Editorial Manager® and ProduXion Manager® from Aries Systems Corporation

Click here to access/download;Manuscript;EUVEN_final.docx

The new COST Action European Venom Network (EUVEN) – synergy and future perspectives of modern venomics

Maria Vittoria Modica [1]*, Rafi Ahmad [2], Stuart Ainsworth [3], Gregor Anderluh [4], Agostinho Antunes [5], Dimitris Beis [6], Figen Caliskan [7], Mauro Dalla Serra [8], Sebastien Dutertre [9], Yehu Moran [10], Ayse Nalbantsoy [11], Naoual Oukkache [12], Stano Pekar [13], Maido Remm [14], Bjoern Marcus von Reumont [15], Yiannis Sarigiannis [16], Andrea Tarallo [17], Jan Tytgat [18], Eivind Andreas Baste Undheim [19], Yuri Utkin [20], Aida Verdes [21], Aude Violette [22], Giulia Zancolli [23]

[1] Department of Biology and Evolution of Marine Organisms, Stazione Zoologica Anton Dohrn, Naples, Italy; mariavittoria.modica@szn.it

[2] Department of Biotechnology, Inland Norway University of Applied Sciences, Hamar, Norway; rafi.ahmad@inn.no

[3] Liverpool School of Tropical Medicine UK; stuart.ainsworth@lstmed.ac.uk

[4] National Institute of Chemistry Ljubljana, Slovenia; gregor.anderluh@ki.si

[5] CIIMAR/CIMAR, Interdisciplinary Centre of Marine and Environmental Research, University of Porto; and Department of Biology, Faculty of Sciences, University of Porto, Porto, Portugal; aantunes@ciimar.up.pt

[6] Biomedical Research Foundation Academy of Athens, Greece; dbeis@bioacademy.gr

[7] Eskişehir Osmangazi University, Biology Department, Eskişehir, Turkey; fcalis@ogu.edu.tr

[8] Istituto di Biofisica, Consiglio Nazionale delle Ricerche, Genova, Italy; mauro.dallaserra@cnr.it

[9] Institut des Biomolécules Max Mousseron, Université de Montpellier, CNRS, ENSCM, 34095 Montpellier, France; sebastien.dutertre@umontpellier.fr

[10] Department of Ecology, Evolution and Behavior, Alexander Silberman Institute of Life Sciences, The Hebrew University of Jerusalem, Jerusalem, Israel; yehu.moran@mail.huji.ac.il

[11] Ege University, Bioengineering Department, Izmir, Turkey; analbantsoy@gmail.com

[12] Institut Pasteur of Morocco, Casablanca; oukkache.naoual@gmail.com

[13] Masaryk university, Brno, Czech Republic; pekar@sci.muni.cz

[14] University of Tartu, Estonia; maido.remm@ut.ee

[15] Justus Liebig University, Giessen, Germany; Bjoern.Von-Reumont@agrar.uni-giessen.de; LOEWE Translational Biodiversity Genomics, Frankfurt, Germany; bmvr@arcor.de

[16] University of Nicosia, Cyprus; sarigiannis.i@unic.ac.cy

[17] Department of Research infrastructures for Marine Biological Resources, Stazione Zoologica Anton Dohrn, Naples, Italy; andrea.tarallo@szn.it

[18] KU Leuven, Belgium; jan.tytgat@kuleuven.be

[19] Centre for Ecological and Evolutionary Synthesis, Department of Biosciences, University of Oslo, Norway; e.a.b.undheim@ibv.uio.no

[20] Shemyakin-Ovchinnikov Institute of Bioorganic Chemistry, Russian Academy of Sciences, Moscow, Russian Federation; yutkin@yandex.ru

[21] Museo Nacional de Ciencias Naturales, Consejo Superior de Investigaciones Científicas, Madrid, Spain; Natural History Museum, London UK; aida.verdes@mncn.csic.es

[22] Alphabiotoxine Laboratory, Montroeul-au-Bois, Belgium; aude.violette@alphabiotoxine.com

[23] Department of Ecology and Evolution, University of Lausanne, Switzerland; giulia.zancolli@gmail.com

* Correspondence: mariavittoria.modica@szn.it;

Abstract: Venom research is a highly multidisciplinary field that involves multiple subfields of biology, informatics, pharmacology, medicine and other areas. These different research facets are often technologically challenging and pursued by different teams lacking connection with each other. This lack of coordination hampers the full development of venom investigation and applications. The COST Action CA19144 – European Venom Network, was recently launched to promote synergistic interactions among different stakeholders and foster venom research at the European level.

Keywords: COST; venom; toxins; networking; interdisciplinarity.

Background

Venomous species represent about 15% of the global estimated animal biodiversity, are omnipresent in aquatic and terrestrial habitats, and evolved independently in all metazoan lineages in more than 100 instances [1]. Venoms are complex mixtures of bioactive compounds, mostly peptides and proteins, that evolved through millions of years of natural selection predominantly for predation and defense. Venom toxins are adaptive and highly convergent traits, extremely useful to understand the evolutionary mechanisms that link genotype, phenotype, and protein function.

In addition, toxins are streamlined to act fast at very low concentrations, being highly specific with key physiological targets of prey and/or predators (ion channels, enzymes and cellular membrane components) [2]. Many toxins target the neuromuscular system, while others possess anticoagulant, cytolytic, anesthetic and hypotensive activities [3]. These characteristics make them ideal candidates for biotechnological applications. Ten animal-derived drugs have been so far approved and several others are in various stages of clinical trials to treat a wide array of diseases

including cancer, hypertension, acute coronary syndromes and chronic pain [4]. Besides medicine, venoms toxins have great potential in other biotechnological fields: spider toxins to develop eco-friendly insecticides and other agrochemicals [4]; ion channel blockers from cone snails and bees for cosmeceutical applications [5]; and pore-forming toxins for sequencing and sensing technologies [6].

Venomics as a multidisciplinary playground

Venom investigation involves many scientific disciplines that in recent years have undergone great technological improvements (Figure 1). These fast-evolving technologies foster venomics research, but also bring new challenges, requiring considerable integrative expertise.

High-throughput techniques have facilitated the characterization of complex venoms even in non-model organisms [7]. Transcriptomic data obtained by latest RNA-Seq technologies are often integrated with bottom-up proteomics, in which high-performance liquid chromatography is coupled with tandem mass spectrometry. This proteo-transcriptomics approach allows the detection of low-copy transcripts and post-translational modifications, and a precise relative quantification of expressed proteins. Integration of genomic data is still uncommon, despite its promise for understanding evolutionary and regulatory patterns of venom compounds.

Bioinformatics pipelines are used for similarity-based screening and identification of promising candidates. Afterwards, the peptide and protein toxins are synthesized via solid-phase peptide synthesis and regioselective folding, or by a variety of different recombinant expression systems to obtain a realistic folding pattern. When separation and isolation of each compound is not achievable due to the low quantity of raw venom, these procedures can yield amounts of proteins suitable for subsequent activity testing, although they require extensive optimization for each component.

Activity screening mostly relies on electrophysiology that is applied on multiple neuroreceptors, ligand-gated and voltage-gated ion channels involved in neurodegenerative and drug dependency disorders, in immune system regulation, anesthesia and neuropathic pain. Electrophysiology also includes *ex vivo* assays and bi-dimensional array assays on tissue preparations for neurological disorders and trauma. Other activity screening tests target hormonal pathways, cancer, cardiovascular or inflammatory disorders; diabetes and obesity, infectious diseases. Bioactivity-driven identification of novel compounds is further tested by *in vivo* phenotypic screens [8].

In addition, biophysical approaches such as, X-ray crystallography, nuclear magnetic resonance spectroscopy, surface plasmon resonance spectroscopy, isothermal titration calorimetry and micro-

computer tomography have become key components of drug discovery platforms and venom systems identification. *In silico* approaches, such as molecular modeling, have also become widely used for studying venom components, providing structural information and theoretical understanding of the molecular mechanisms of toxin action [9].

The COST Action EUVEN: from fragmentation to integration

The different facets of venomics are typically pursued by different research groups, whose level of collaboration in EU is not adequate to face the increasing challenges in venomics research, as reflected by the decreased relative contributions of EU scientists to global venom research in the last 20 years (Figure 2).

The European Venom Network COST Action CA19144 (EUVEN) (https://euven-network.eu) was launched in October 2020 to promote an efficient exchange of ideas, knowledge, benchmarks, and to involve all relevant stakeholders to foster European venom research. The European Cooperation in Science and Technology (COST) Association operates since 1971 funding bottom-up networks that run for four years to boost research, innovation and careers through a series of collaborative activities including workshops, conferences, working group meetings, training schools, short-term scientific missions, dissemination and outreach.

In bringing together experts from all relevant fields, EUVEN develops protocols on best practices in venomics, and identifies the most promising novel technological tools, animal models and untapped physiological targets to be integrated into current venom research. Stronger collaborations with non-academic stakeholders, especially with small and medium enterprises (SMEs) are enforced as a prerequisite for promoting biomedical, diagnostic, agrochemical, cosmeceutical or nanobiotechnological applications of venom compounds. EUVEN aims at involving also non-professional societies to foster biodiversity-based research, with the support of natural history museums. Our major research and capacity-building objectives will be tackled through the engagement of participants (currently from 31 countries) in five working groups (WGs).

WG1: Novel targets in venom research – In this WG, novel targets are established and validated to expand and diversify the current main focus of the research community on just a few model organisms, diseases and molecular targets.

WG2: Best practices and innovative tools – The aim of this WG is to validate and develop best research practices to minimize variation and gather reproducible and comparable results, in all

experimental and analytical steps contributing to venom investigation. Additionally, the integration of innovative tools in venom research will be evaluated.

WG3: Interaction with industry – The focus of WG3 is the interplay between academic researchers and industrial partners to transform discoveries and knowledge of researchers into innovations for society. EUVEN supports different initiatives to encourage participation of SMEs in meetings and discussions, and increase the appeal of venom research for industries.

WG4: Web resources – Available web resources for venom research currently lack a streamlined synergism. WG4 aims at integrating databases in a single repository that will also implement the most useful computational analytical tools for the detection of relevant features, including folds/activities of potential applicative interest.

WG5: Training – Given the multidisciplinary nature of venom research, EUVEN plans to offer extensive training opportunities in all relevant venom research disciplines.

Conclusion

The new COST Action EUVEN provides a flexible platform for scientists to overcome the lack of coordination, tools, and resources, and develop a fully synergistic network. To guarantee the coverage of the diverse topics of interest in EUVEN, and build an effective network across Europe and beyond, it is fundamental to engage the broadest participation possible from all COST participating countries. Near-neighbor and international partner countries can also request to join EUVEN, and participate in networking activities.

We believe that building an effective network, able to bridge different scientific disciplines and sectors, constitutes a fundamental prerequisite to fully develop the extraordinary transformative potential of venom research.

Figures

Figure 1. The multidisciplinary nature of venom investigation, including its connection with different research fields, and main drivers that are affecting it.

Figure 2. Number of indexed publications on venom between the years 2000 and 2020. The figure allows the comparison between the EU and extra-EU authors contributions to venom research. The non-EU authored publications doubled in the last 20 years. By contrast, the EU-only authored

ones only increased about 20%. The word “venom” has been submitted as query for “topic”. EU- and non-EU countries were then excluded to obtain publications lists of extra-EU authors only and EU-authors only, respectively (Source Web of Science, last updated December 2020).

Author Contributions: Conceptualization, M.V.M., A.A., G.A., S.D., B.M.v.R., and S.P.; writing—original draft preparation, M.V.M.; figures preparation, B.M.v.R., A.T.; writing—review and editing, all authors.; project administration, M.V.M, G.A., A.T. Except for the first, authors are listed alphabetically with respect to last name. All authors have read and agreed to the published version of the manuscript. Parts of this paper are derived from the Memorandum of Understanding for the implementation of the COST Action “European Venom Network” (EUVEN) CA19144.

Funding: The authors acknowledge support from the European Cooperation in Science and Technology (COST) through the Action CA19144 EUVEN.

Conflicts of Interest: The authors declare no conflict of interest.

References

1. Schendel V, Rash LD, Jenner RA, Undheim EAB. The Diversity of Venom: The Importance of Behavior and Venom System Morphology in Understanding Its Ecology and Evolution. *Toxins* 2019; 11(11): 666, doi: 10.3390/toxins11110666

2. Barua A, Mikheyev AS, Russo C. Many Options, Few Solutions: Over 60 My Snakes Converged on a Few Optimal Venom Formulations. *Mol. Biol. Evol.* 2019, 36:1964–1974, doi:10.1093/molbev/msz125.

3. Fry BG, Roelants K, Champagne DE, et al. The Toxicogenomic Multiverse: Convergent Recruitment of Proteins Into Animal Venoms. *Annu. Rev. Genomics Hum. Genet.* 2009; 10: 483–511, doi:10.1146/annurev.genom.9.081307.164356.

4. Holford M, Daly M, King GF, Norton RS Venoms to the rescue. Science, 2018; 361(6405):

842-844. DOI: 10.1126/science.aau7761

5. Kim H, Park SY, Lee G. Potential therapeutic applications of bee venom on skin disease and its mechanisms: A literature review. *Toxins,* 2019; 11:374.

6. Morante K, Bellomio A, Viguera AR, González-Mañas JM, Tsumoto K, Caaveiro JMM. The isolation of new pore-forming toxins from the sea anemone *Actinia fragacea* provides insights into the mechanisms of actinoporin evolution. *Toxins,* 2019;11: 401. doi:10.3390/toxins11070401.

7. Drukewitz SH, von Reumont BM. The Significance of Comparative Genomics in Modern Evolutionary Venomics. *Front. Ecol. Evol.* 2019; 7:163, doi:10.3389/fevo.2019.00163.

8. Vargas RA, Sarmiento K, Vásquez IC. Zebrafish (*Danio rerio*): A Potential Model for Toxinological Studies. *Zebrafish* 2015; 12:320–326, doi:10.1089/zeb.2015.1102.

9. Leffler AE, Kuryatov A, Zebroski HA, et al. Discovery of peptide ligands through docking and virtual screening at nicotinic acetylcholine receptor homology models. *Proc. Natl. Acad. Sci.* 2017;114:E8100–E8109, doi:10.1073/pnas.1703952114.

10. World Economic Forum: These life-saving drugs are made from deadly venom Available online: https://www.weforum.org/agenda/2018/11/venomics-deadly-toxins-life-saving-drugs-mande-holford/ (accessed on Dec 20, 2020).

Figure1

Click here to access/download;Figure;Figure1.jpg

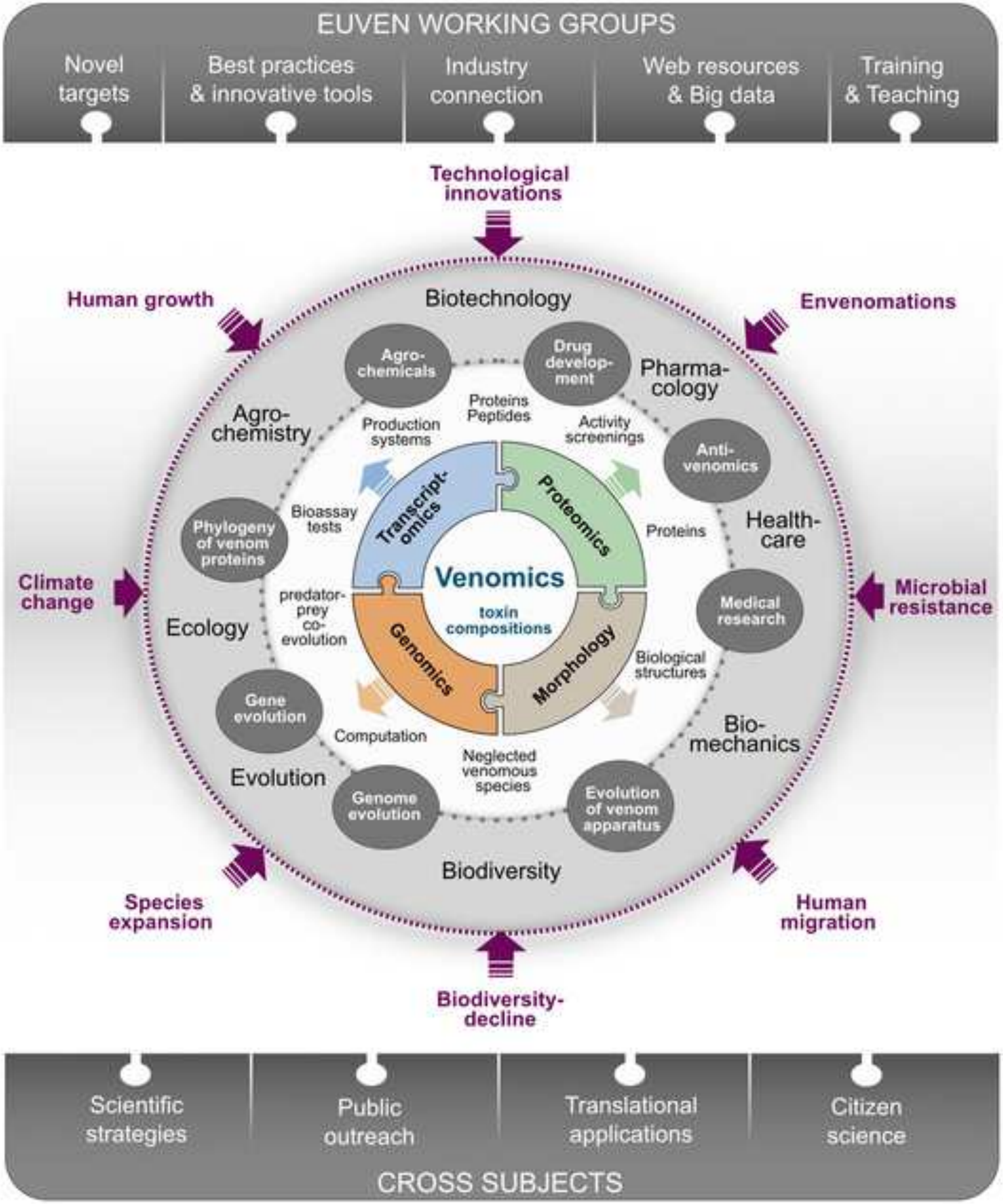

Figure2

Click here to access/download;Figure;Figure2.png

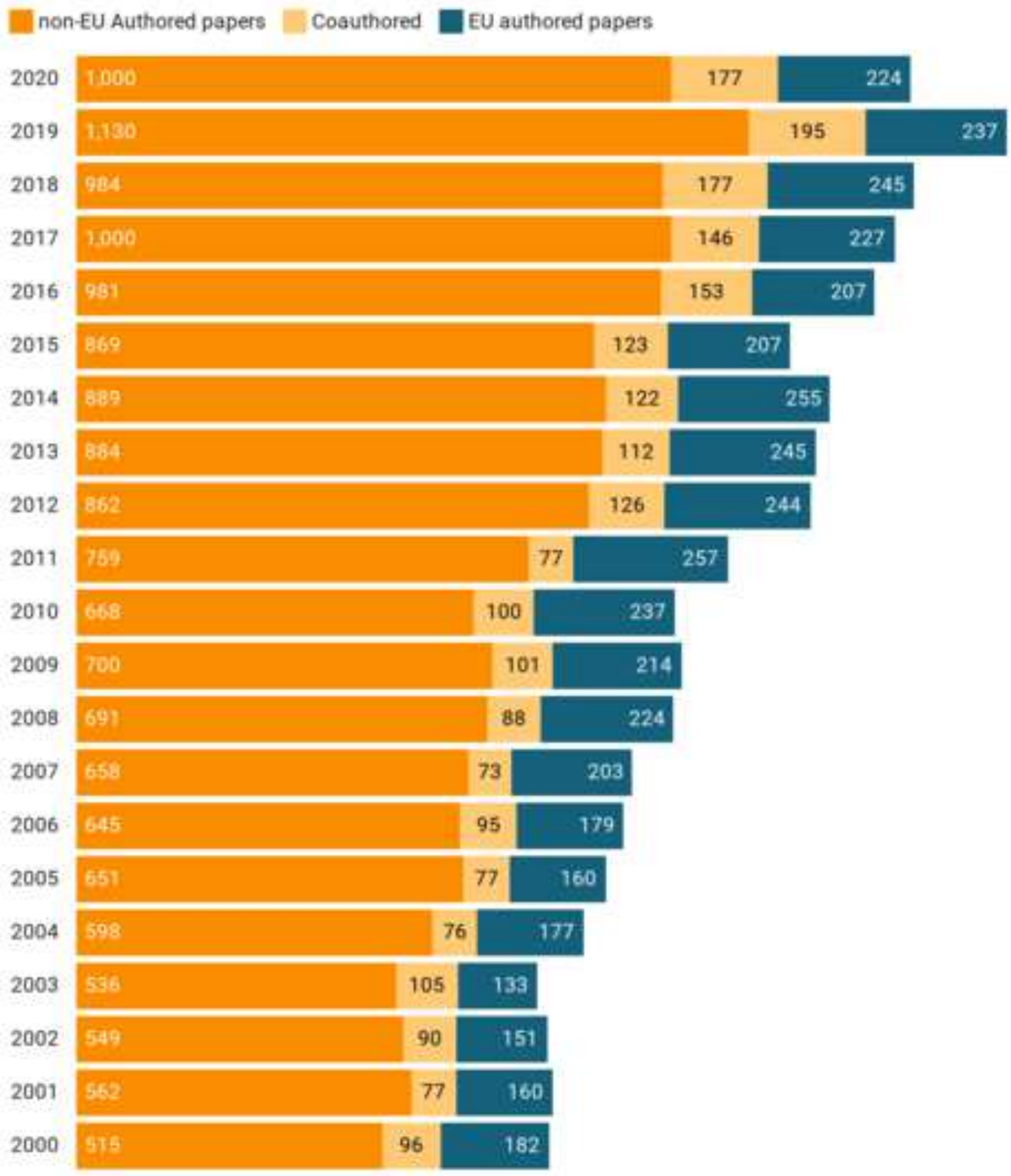

Supplement: giab019_GIGA-D-21-00035_Original_Submission [file giab019_giga-d-21-00035_original_submission.pdf]
