# Supplementary material for: The new COST Action European Venom Network (EUVEN)—synergy and future perspectives of modern venomics
Source: Gigascience. 2021 Mar 25;10(3):giab019. doi: 10.1093/gigascience/giab019 (PMC7992391; doi:10.1093/gigascience/giab019)

GigaScience

The new COST Action European Venom Network (EUVEN) – synergy and future perspectives of modern venomics

--Manuscript Draft--

**Manuscript Number:**	GIGA-D-21-00035R1
**Full Title:**	The new COST Action European Venom Network (EUVEN) – synergy and future perspectives of modern venomics
**Article Type:**	Commentary
**Funding Information:**	European Cooperation in Science and Technology (COST) (CA19144) — Not applicable
**Abstract:**	Venom research is a highly multidisciplinary field that involves multiple subfields of biology, informatics, pharmacology, medicine and other areas. These different research facets are often technologically challenging and pursued by different teams lacking connection with each other. This lack of coordination hampers the full development of venom investigation and applications. The COST Action CA19144 – European Venom Network, was recently launched to promote synergistic interactions among different stakeholders and foster venom research at the European level.
**Corresponding Author:**	Maria Vittoria Modica, Ph.D.<br>Stazione Zoologica Anton Dohrn<br>Napoli, ITALY
**Corresponding Author Secondary Information:**	
**Corresponding Author's Institution:**	Stazione Zoologica Anton Dohrn
**Corresponding Author's Secondary Institution:**	
**First Author:**	Maria Vittoria Modica
**First Author Secondary Information:**	
**Order of Authors:**	Maria Vittoria Modica
	Rafi Ahmad
	Stuart Ainsworth
	Gregor Anderluh
	Agostinho Antunes
	Dimitri Beis
	Figen Caliskan
	Mauro Dalla Serra
	Sebastien Dutertre
	Yehu Moran
	Ayse Nalbantsoy
	Naoual Oukkache
	Stano Pekar
	Maido Remm
	Bjoern Marcus von Reumont
	Yiannis Sarigiannis
	Andrea Tarallo

Powered by Editorial Manager® and ProduXion Manager® from Aries Systems Corporation

	Jan Tytgat
	Yuri Utkin
	Aida Verdes
	Aude Violette
	Giulia Zancolli
**Order of Authors Secondary Information:**	
**Response to Reviewers:**	Reviewer #1: This manuscript informs the scientific community, particularly those researchers in the field of molecular toxinology, of the launch of the COST initiative "Action CA19144 - European Venom Network", aimed at promoting synergistic interactions among different stakeholders and foster venom research at the European level. This announcement is clearly formulated and the content and objectives of the COST action discussed. Nothing to criticize in this regard. My only criticism of the content of the manuscript is of Figure 2. I just don't see any point in highlighting a comparison between the EU and extra-EU authors contributions to venom research just referring to the number of indexed publications on venom between the years 2000 and 2020. Number of publications does not tell anything about the global quality of the research! As an example of a serious and informative bibliometric analysis, I would recommend authors to read Groneberg DA, Geier V, KlingelhoÈfer D,Gerber A, Kuch U, Kloft B (2016) Snakebite Envenoming- A Combined Density Equalizing Mapping and Scientometric Analysis of the Publication History. PLoS Negl Trop Dis 10(11): e0005046. doi:10.1371/journal.pntd.0005046.<br><br>Authors’ response:<br>The application of bibliometric indicators for assessing scientific performance has always been a controversial issue, and indeed the exhaustive approach used by Groneberg et al. for evaluating publications on a subtopic of venom research is an important step forward. However, such a comprehensive scientometric analysis is beyond the scope of our commentary. The number of publications in peer-reviewed journals over a certain time period is a common and intuitive way to broadly assess the involvement of the research community in a given topic, which is exactly our aim in this commentary. We would instead refrain here from evaluating the quality of European venom research as a whole, since its complexity (due to its recognized multidisciplinarity) would require extensive effort and better fit a separate publication. However, the lack of a qualitative scientometric evaluation of venom research output suggests that such a broad-scale effort may indeed constitute an additional goal of the Action itself, and we will take this possibility in the due consideration along its implementation.<br><br>It would also be desirable to refer to web page links where an interested reader could find all the documentation on this Cost Action, including information on the participants and the detailed objectives of the project.<br><br>Authors response:<br>The reference to the Action website, where all relevant information can be easily found, is provided at the beginning of the paragraph The COST Action EUVEN: from fragmentation to integration<br><br>*********<br>Reviewer #2: The commentary article The new COST Action European Venom Network (EUVEN) - synergy and future perspectives of modern venomics describes the multidisciplinary nature of venom research and presents the EUVEN, a new EU COST action, as a means of uniting stakeholders in distinct fields. The manuscript presents a brief summary of recent and on-going developments in venom research, highlighting the multi-faceted nature of modern venom research. The authors specifically note the utility of high-throughput molecular characterization and quantification methods (e.g. RNAseq, qMS), the integration of bioinformatic techniques for data processing, and biophysical and in silico approaches for studying molecular characteristics and modes of toxin action. The authors then introduce EUVEN as a research network with the aim of strengthening collaboration and generating synergy among traditionally disparate venom research groups in the EU. Working as a collective, EUVEN will establish best practices in venom research as well as identify

and integrate emerging technologies. EUVEN will operate through five working groups focused on target outcomes such as building interaction with industry and multidisciplinary training. The article concludes that EUVEN will provide the resources to develop synergy among participants and stakeholders for transformative work in venom research.

I found the article well written and well-organized. The background provided is appropriate and describes the multidisciplinary nature of the field which presently challenges venom research groups that have traditionally focused on specific research areas. The second half of the article describes how EUVEN will address these challenges. Most of my comments are very minor and provide some suggestions about grammar and wording. I would encourage the authors to revisit Figure 1 somewhat. Figure 1 is a very nice-looking figure and in the broad sense fairly intuitive -- when I look at the figure it conveys that venom research is multifaceted and involves many different fields/subfields. However, when I started trying to interpret different parts of the figure things became less clear to me. Specifically, it is not entirely apparent to me what the connections are between different parts of the figure. Do all arrows indicate 'drivers', such that interior arrows are methodological drivers and exterior arrows external drivers? Similarly, how would the authors classify the white ring, grey ring, and grey circles in the main circle? Fields and subfields? Finally, it is not clear to me exactly what the roles of the grey rectangles at the top of the bottom of the figure are. I realize these fields indicate the working groups and cross subjects, but I am not sure how they link to the rest of the figure as there does not seem to be any correspondence with main parts of

the circle—at present it seems like they could be removed with no effect on the message the figure conveys. I believe having a little more information on how this figure is organized would greatly enrich its interpretation and utility. I believe most of the relevant information can be provided in the figure caption, though perhaps the authors could convey this information in a figure legend or something if they prefer.

I have included my other minor comments below and on the included pdf (comments are the same here and on the pdf). I commend the authors on a well-constructed manuscript, and I look forward to both seeing the manuscript in press and the future work and outcomes of EUVEN more broadly.

Authors' response:

We have modified the figure caption as follows in order to convey the required information, explicitly describing the conceptual links between the different parts of the figure.

"The multidisciplinary, integrative and interconnected vision of venom research proposed in EUVEN. The centre is composed of modern morphology and -omics methods, in particular proteomics, transcriptomics and genomics. In the surrounding white circle, main aspects of current venom research are indicated and summarized by the major topics in the oval grey circles. These major topics are loosely associated with broader themes given in the grey circle. The whole system is affected and interacts with outer drivers (in purple). This integrative scheme is the heart of EUVEN, in which five major Working Groups focus on these topics and methods (top grey bar; see text for details). The cross subjects in which the scientific, technological and socio-economic impact of EUVEN will be realized are outlined in the lower grey bar."

-Andrew Mason

Minor comments:

Page 2, line 9 (under Background), First sentence of paragraph 2: I just wanted to verify that Barua et al. 2019 is citation that the authors intended to use here.

My reading of Barua et al. 2019 is that it does not provide direct evidence of venoms being fast acting at low concentrations or being physiologically specific, but rather presents evidence of adaptive convergence in snakes. To me, this citation would be better suited to supporting the previous sentence ('Venom toxins are adaptive and highly convergent traits...') while something like the Casewell et al. 2013 'Complex cocktails' review, which includes a summary of some evidence for prey specificity in venom, would better support this sentence.

Authors' response:

The reference suggested by the reviewer is indeed more appropriate. We have replaced Barua et al. with Casewell et al.

Powered by Editorial Manager® and ProduXion Manager® from Aries Systems Corporation

Page 2, line 11 (under Background), "Ten animal derived drugs have been so far approved": The placement of 'so far' reads just a bit awkwardly to me, though I do not think it is grammatically incorrect. I would write the sentence as 'Ten animal-derived drugs so far have been approved' or even 'To date, ten animal-derived drugs have been approved and ...'
Authors' response:
We have modified the text according to the reviewer's suggestion.

Page 4, line 14, "The European Cooperation in Science and Technology (COST) Association operates": change 'operates' to 'has operated'
Authors' response:
We have modified the text according to the reviewer's suggestion.

Page 4, line 23, "EUVEN aims at involving also": change to "EUVEN also aims to involve"
Authors' response:
We have modified the text according to the reviewer's suggestion.

Page 4, line 24: remove comma after "biodiversity-based research"
Authors' response:
We have modified the text according to the reviewer's suggestion.

Page 4, line 31: remove comma after "comparable results"
Authors' response:
We have modified the text according to the reviewer's suggestion.

Page 5, line 4, "transform discoveries and knowledge of researchers into": I would recommend changing "knowledge of researchers" to either "research knowledge" or "researchers' knowledge" depending on what the authors would like to convey.
Authors' response:
We have modified the text according to the reviewer's suggestion.

Page 5, line 14, first sentence under Conclusion, to overcome the lack of coordination, tools, and resources, and develop a fully synergistic network.: think there is something a bit off about the wording here. It seems like 'develop a fully synergistic network' should either be presented as an outcome (as in "to overcome the lack of coordination, tools, and resources to develop a fully synergistic network") or as a means of accomplishment (as in "to overcome the lack of coordination, tools, and resources through the development of a fully synergistic network"). But that could just be how I was expecting the sentence to be structured and perhaps as it is written is exactly what the authors wanted to convey.
Authors' response:
We have modified the text according to the reviewer's suggestion.

Page 5, line 20, "building an effective network, able to bridge": an effective network that is able to bridge
Authors' response:
We have modified the text according to the reviewer's suggestion.

Page 5, line 21: remove comma after sector
Authors' response:
We have modified the text according to the reviewer's suggestion.

Page 5, line 21, extraordinary: should be extraordinarily
Authors' response:
We have modified the text according to the reviewer's suggestion.

Figure 2 legend, to obtain publications lists: to obtain publication lists
Authors' response:
We have modified the text according to the reviewer's suggestion.

References, 10: I don't believe that there was a 10th citation in the text.
Authors' response:

Powered by Editorial Manager® and ProduXion Manager® from Aries Systems Corporation

	We have removed this reference.
Additional Information:	
Question	Response
Are you submitting this manuscript to a special series or article collection?	No
**Experimental design and statistics**<br><br>Full details of the experimental design and statistical methods used should be given in the Methods section, as detailed in our Minimum Standards Reporting Checklist. Information essential to interpreting the data presented should be made available in the figure legends.<br><br>Have you included all the information requested in your manuscript?	No
If not, please give reasons for any omissions below.<br><br>as follow-up to "**Experimental design and statistics**<br><br>Full details of the experimental design and statistical methods used should be given in the Methods section, as detailed in our Minimum Standards Reporting Checklist. Information essential to interpreting the data presented should be made available in the figure legends.<br><br>Have you included all the information requested in your manuscript?<br><br>"	Not applicable
**Resources**<br><br>A description of all resources used, including antibodies, cell lines, animals and software tools, with enough information to allow them to be uniquely identified, should be included in the Methods section. Authors are strongly	No

encouraged to cite Research Resource Identifiers (RRIDs) for antibodies, model organisms and tools, where possible.<br><br>Have you included the information requested as detailed in our Minimum Standards Reporting Checklist?	
If not, please give reasons for any omissions below.<br><br>as follow-up to "**Resources**<br><br>A description of all resources used, including antibodies, cell lines, animals and software tools, with enough information to allow them to be uniquely identified, should be included in the Methods section. Authors are strongly encouraged to cite Research Resource Identifiers (RRIDs) for antibodies, model organisms and tools, where possible.<br><br>Have you included the information requested as detailed in our Minimum Standards Reporting Checklist?<br><br>"	Not appplicable
**Availability of data and materials**<br><br>All datasets and code on which the conclusions of the paper rely must be either included in your submission or deposited in publicly available repositories (where available and ethically appropriate), referencing such data using a unique identifier in the references and in the “Availability of Data and Materials” section of your manuscript.<br><br>Have you have met the above requirement as detailed in our Minimum Standards Reporting Checklist?	No
If not, please give reasons for any	Not applicable

omissions below.<br><br>as follow-up to "**Availability of data and materials**<br><br>All datasets and code on which the conclusions of the paper rely must be either included in your submission or deposited in publicly available repositories (where available and ethically appropriate), referencing such data using a unique identifier in the references and in the "Availability of Data and Materials" section of your manuscript.<br><br>Have you have met the above requirement as detailed in our Minimum Standards Reporting Checklist?<br><br>"	

Powered by Editorial Manager® and ProduXion Manager® from Aries Systems Corporation

main text Click here to access/download;Manuscript;EUVEN_resub.docx

The new COST Action European Venom Network (EUVEN) – synergy and future perspectives of modern venomics

Maria Vittoria Modica [1]*, Rafi Ahmad [2], Stuart Ainsworth [3], Gregor Anderluh [4], Agostinho Antunes [5], Dimitris Beis [6], Figen Caliskan [7], Mauro Dalla Serra [8], Sebastien Dutertre [9], Yehu Moran [10], Ayse Nalbantsoy [11], Naoual Oukkache [12], Stano Pekar [13], Maido Remm [14], Bjoern Marcus von Reumont [15], Yiannis Sarigiannis [16], Andrea Tarallo [17], Jan Tytgat [18], Eivind Andreas Baste Undheim [19], Yuri Utkin [20], Aida Verdes [21], Aude Violette [22], Giulia Zancolli [23]

[1] Department of Biology and Evolution of Marine Organisms, Stazione Zoologica Anton Dohrn, Naples, Italy; mariavittoria.modica@szn.it

[2] Department of Biotechnology, Inland Norway University of Applied Sciences, Hamar, Norway; rafi.ahmad@inn.no

[3] Liverpool School of Tropical Medicine UK; stuart.ainsworth@lstmed.ac.uk

[4] National Institute of Chemistry Ljubljana, Slovenia; gregor.anderluh@ki.si

[5] CIIMAR/CIMAR, Interdisciplinary Centre of Marine and Environmental Research, University of Porto; and Department of Biology, Faculty of Sciences, University of Porto, Porto, Portugal; aantunes@ciimar.up.pt

[6] Biomedical Research Foundation Academy of Athens, Greece; dbeis@bioacademy.gr

[7] Eskişehir Osmangazi University, Biology Department, Eskişehir, Turkey; fcalis@ogu.edu.tr

[8] Istituto di Biofisica, Consiglio Nazionale delle Ricerche, Genova, Italy; mauro.dallaserra@cnr.it

[9] Institut des Biomolécules Max Mousseron, Université de Montpellier, CNRS, ENSCM, 34095 Montpellier, France; sebastien.dutertre@umontpellier.fr

[10] Department of Ecology, Evolution and Behavior, Alexander Silberman Institute of Life Sciences, The Hebrew University of Jerusalem, Jerusalem, Israel; yehu.moran@mail.huji.ac.il

[11] Ege University, Bioengineering Department, Izmir, Turkey; analbantsoy@gmail.com

[12] Institut Pasteur of Morocco, Casablanca; oukkache.naoual@gmail.com

[13] Masaryk university, Brno, Czech Republic; pekar@sci.muni.cz

[14] University of Tartu, Estonia; maido.remm@ut.ee

[15] Justus Liebig University, Giessen, Germany; Bjoern.Von-Reumont@agrar.uni-giessen.de; LOEWE Translational Biodiversity Genomics, Frankfurt, Germany; bmvr@arcor.de

[16] University of Nicosia, Cyprus; sarigiannis.i@unic.ac.cy

[17] Department of Research infrastructures for Marine Biological Resources, Stazione Zoologica Anton Dohrn, Naples, Italy; andrea.tarallo@szn.it

[18] KU Leuven, Belgium; jan.tytgat@kuleuven.be

[19] Centre for Ecological and Evolutionary Synthesis, Department of Biosciences, University of Oslo, Norway; e.a.b.undheim@ibv.uio.no

[20] Shemyakin-Ovchinnikov Institute of Bioorganic Chemistry, Russian Academy of Sciences, Moscow, Russian Federation; yutkin@yandex.ru

[21] Museo Nacional de Ciencias Naturales, Consejo Superior de Investigaciones Científicas, Madrid, Spain; Natural History Museum, London UK; aida.verdes@mncn.csic.es

[22] Alphabiotoxine Laboratory, Montroeul-au-Bois, Belgium; aude.violette@alphabiotoxine.com

[23] Department of Ecology and Evolution, University of Lausanne, Switzerland; giulia.zancolli@gmail.com

* Correspondence: mariavittoria.modica@szn.it;

Abstract: Venom research is a highly multidisciplinary field that involves multiple subfields of biology, informatics, pharmacology, medicine and other areas. These different research facets are often technologically challenging and pursued by different teams lacking connection with each other. This lack of coordination hampers the full development of venom investigation and applications. The COST Action CA19144 – European Venom Network, was recently launched to promote synergistic interactions among different stakeholders and foster venom research at the European level.

Keywords: COST; venom; toxins; networking; interdisciplinarity.

Background

Venomous species represent about 15% of the global estimated animal biodiversity, are omnipresent in aquatic and terrestrial habitats, and evolved independently in all metazoan lineages in more than 100 instances [1]. Venoms are complex mixtures of bioactive compounds, mostly peptides and proteins, that evolved through millions of years of natural selection predominantly for predation and defense. Venom toxins are adaptive and highly convergent traits, extremely useful to understand the evolutionary mechanisms that link genotype, phenotype, and protein function.

In addition, toxins are streamlined to act fast at very low concentrations, being highly specific with key physiological targets of prey and/or predators (ion channels, enzymes and cellular membrane components) [2]. Many toxins target the neuromuscular system, while others possess anticoagulant, cytolytic, anesthetic and hypotensive activities [3]. These characteristics make them ideal candidates for biotechnological applications. To date, ten animal-derived drugs have been approved and several others are in various stages of clinical trials to treat a wide array of diseases

including cancer, hypertension, acute coronary syndromes and chronic pain [4]. Besides medicine, venoms toxins have great potential in other biotechnological fields: spider toxins to develop eco-friendly insecticides and other agrochemicals [4]; ion channel blockers from cone snails and bees for cosmeceutical applications [5]; and pore-forming toxins for sequencing and sensing technologies [6].

Venomics as a multidisciplinary playground

Venom investigation involves many scientific disciplines that in recent years have undergone great technological improvements (Figure 1). These fast-evolving technologies foster venomics research, but also bring new challenges, requiring considerable integrative expertise.

High-throughput techniques have facilitated the characterization of complex venoms even in non-model organisms [7]. Transcriptomic data obtained by latest RNA-Seq technologies are often integrated with bottom-up proteomics, in which high-performance liquid chromatography is coupled with tandem mass spectrometry. This proteo-transcriptomics approach allows the detection of low-copy transcripts and post-translational modifications, and a precise relative quantification of expressed proteins. Integration of genomic data is still uncommon, despite its promise for understanding evolutionary and regulatory patterns of venom compounds.

Bioinformatics pipelines are used for similarity-based screening and identification of promising candidates. Afterwards, the peptide and protein toxins are synthesized via solid-phase peptide synthesis and regioselective folding, or by a variety of different recombinant expression systems to obtain a realistic folding pattern. When separation and isolation of each compound is not achievable due to the low quantity of raw venom, these procedures can yield amounts of proteins suitable for subsequent activity testing, although they require extensive optimization for each component.

Activity screening mostly relies on electrophysiology that is applied on multiple neuroreceptors, ligand-gated and voltage-gated ion channels involved in neurodegenerative and drug dependency disorders, in immune system regulation, anesthesia and neuropathic pain. Electrophysiology also includes *ex vivo* assays and bi-dimensional array assays on tissue preparations for neurological disorders and trauma. Other activity screening tests target hormonal pathways, cancer, cardiovascular or inflammatory disorders; diabetes and obesity, infectious diseases. Bioactivity-driven identification of novel compounds is further tested by *in vivo* phenotypic screens [8].

In addition, biophysical approaches such as, X-ray crystallography, nuclear magnetic resonance spectroscopy, surface plasmon resonance spectroscopy, isothermal titration calorimetry and micro-

computer tomography have become key components of drug discovery platforms and venom systems identification. *In silico* approaches, such as molecular modeling, have also become widely used for studying venom components, providing structural information and theoretical understanding of the molecular mechanisms of toxin action [9].

The COST Action EUVEN: from fragmentation to integration

The different facets of venomics are typically pursued by different research groups, whose level of collaboration in EU is not adequate to face the increasing challenges in venomics research, as reflected by the decreased relative contributions of EU scientists to global venom research in the last 20 years (Figure 2).

The European Venom Network COST Action CA19144 (EUVEN) (https://euven-network.eu) was launched in October 2020 to promote an efficient exchange of ideas, knowledge, benchmarks, and to involve all relevant stakeholders to foster European venom research. The European Cooperation in Science and Technology (COST) Association has operated since 1971 funding bottom-up networks that run for four years to boost research, innovation and careers through a series of collaborative activities including workshops, conferences, working group meetings, training schools, short-term scientific missions, dissemination and outreach.

In bringing together experts from all relevant fields, EUVEN develops protocols on best practices in venomics, and identifies the most promising novel technological tools, animal models and untapped physiological targets to be integrated into current venom research. Stronger collaborations with non-academic stakeholders, especially with small and medium enterprises (SMEs) are enforced as a prerequisite for promoting biomedical, diagnostic, agrochemical, cosmeceutical or nanobiotechnological applications of venom compounds. EUVEN also aims to involve non-professional societies to foster biodiversity-based research with the support of natural history museums. Our major research and capacity-building objectives will be tackled through the engagement of participants (currently from 31 countries) in five working groups (WGs).

WG1: Novel targets in venom research – In this WG, novel targets are established and validated to expand and diversify the current main focus of the research community on just a few model organisms, diseases and molecular targets.

WG2: Best practices and innovative tools – The aim of this WG is to validate and develop best research practices to minimize variation and gather reproducible and comparable results in all

experimental and analytical steps contributing to venom investigation. Additionally, the integration of innovative tools in venom research will be evaluated.

WG3: Interaction with industry – The focus of WG3 is the interplay between academic researchers and industrial partners to transform researcher's discoveries and knowledge into innovations for society. EUVEN supports different initiatives to encourage participation of SMEs in meetings and discussions, and increase the appeal of venom research for industries.

WG4: Web resources – Available web resources for venom research currently lack a streamlined synergism. WG4 aims at integrating databases in a single repository that will also implement the most useful computational analytical tools for the detection of relevant features, including folds/activities of potential applicative interest.

WG5: Training – Given the multidisciplinary nature of venom research, EUVEN plans to offer extensive training opportunities in all relevant venom research disciplines.

Conclusion

The new COST Action EUVEN provides a flexible platform for scientists to overcome the lack of coordination, tools, and resources, through the development of a fully synergistic network. To guarantee the coverage of the diverse topics of interest in EUVEN, and build an effective network across Europe and beyond, it is fundamental to engage the broadest participation possible from all COST participating countries. Near-neighbor and international partner countries can also request to join EUVEN, and participate in networking activities.

We believe that building an effective network that is able to bridge different scientific disciplines and sectors constitutes a fundamental prerequisite to fully develop the extraordinarily transformative potential of venom research.

Figures

Figure 1. The multidisciplinary, integrative and interconnected vision of venom research proposed in EUVEN. The centre is composed of modern morphology and omics-methods, in particular proteomics, transcriptomics and genomics. In the surrounding white circle, main aspects of current venom research are indicated and summarized by the major topics in the oval grey circles. These major topics are loosely associated with broader themes given in the grey circle. The whole system

is affected and interacts with outer drivers (in purple). This integrative scheme is the heart of EUVEN, in which five major Working Groups focus on these topics and methods (top grey bar; see text for details). The cross subjects in which the scientific, technological and socio-economic impact of EUVEN will be realized are outlined in the lower grey bar.

Figure 2. Number of indexed publications on venom between the years 2000 and 2020. The figure allows the comparison between the EU and extra-EU authors contributions to venom research. The non-EU authored publications doubled in the last 20 years. By contrast, the EU-only authored ones only increased about 20%. The word "venom" has been submitted as query for "topic". EU- and non-EU countries were then excluded to obtain publication lists of extra-EU authors only and EU-authors only, respectively (Source Web of Science, last updated December 2020).

Author Contributions: Conceptualization, M.V.M., A.A., G.A., S.D., B.M.v.R., and S.P.; writing—original draft preparation, M.V.M.; figures preparation, B.M.v.R., A.T.; writing—review and editing, all authors.; project administration, M.V.M, G.A., A.T. Except for the first, authors are listed alphabetically with respect to last name. All authors have read and agreed to the published version of the manuscript. Parts of this paper are derived from the Memorandum of Understanding for the implementation of the COST Action "European Venom Network" (EUVEN) CA19144.

Funding: The authors acknowledge support from the European Cooperation in Science and Technology (COST) through the Action CA19144 EUVEN.

Conflicts of Interest: The authors declare no conflict of interest.

References

1. Schendel V, Rash LD, Jenner RA, Undheim EAB. The Diversity of Venom: The Importance of Behavior and Venom System Morphology in Understanding Its Ecology and Evolution. *Toxins* 2019; 11(11): 666, doi: 10.3390/toxins11110666

2. Casewell NR, Wüster W, Vonk FJ, Harrison RA, Fry, BG. Complex cocktails: the evolutionary novelty of venoms. *Trends Ecol. Evol.* 2013; 28(4): 219-229, doi:

10.1016/j.tree.2012.10.020

3. Fry BG, Roelants K, Champagne DE, et al. The Toxicogenomic Multiverse: Convergent Recruitment of Proteins Into Animal Venoms. *Annu. Rev. Genomics Hum. Genet.* 2009; 10: 483–511, doi:10.1146/annurev.genom.9.081307.164356.

4. Holford M, Daly M, King GF, Norton RS Venoms to the rescue. Science, 2018; 361(6405): 842-844. DOI: 10.1126/science.aau7761

5. Kim H, Park SY, Lee G. Potential therapeutic applications of bee venom on skin disease and its mechanisms: A literature review. *Toxins,* 2019; 11:374.

6. Morante K, Bellomio A, Viguera AR, González-Mañas JM, Tsumoto K, Caaveiro JMM. The isolation of new pore-forming toxins from the sea anemone *Actinia fragacea* provides insights into the mechanisms of actinoporin evolution. *Toxins,* 2019;11: 401. doi:10.3390/toxins11070401.

7. Drukewitz SH, von Reumont BM. The Significance of Comparative Genomics in Modern Evolutionary Venomics. *Front. Ecol. Evol.* 2019; 7:163, doi:10.3389/fevo.2019.00163.

8. Vargas RA, Sarmiento K, Vásquez IC. Zebrafish (*Danio rerio*): A Potential Model for Toxinological Studies. *Zebrafish* 2015; 12:320–326, doi:10.1089/zeb.2015.1102.

9. Leffler AE, Kuryatov A, Zebroski HA, et al. Discovery of peptide ligands through docking and virtual screening at nicotinic acetylcholine receptor homology models. *Proc. Natl. Acad. Sci.* 2017;114:E8100–E8109, doi:10.1073/pnas.1703952114.

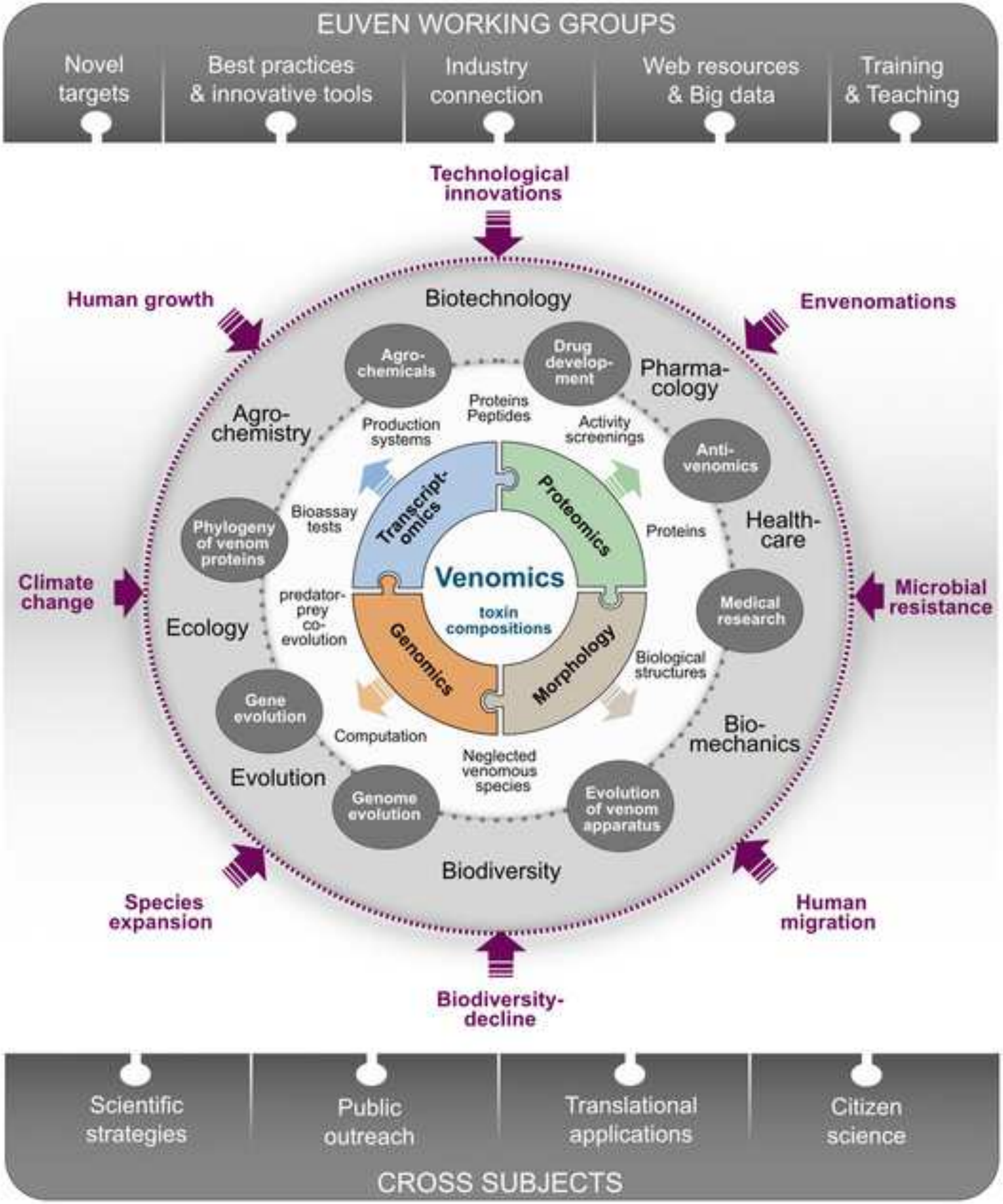

EUVEN WORKING GROUPS
Novel targets
Best practices & innovative tools
Industry connection
Web resources & Big data
Training & Teaching
Technological innovations
Human growth
Envenomations
Climate change
Microbial resistance
Species expansion
Human migration
Biodiversity-decline
Biotechnology
Pharma-cology
Health-care
Bio-mechanics
Biodiversity
Evolution
Ecology
Agro-chemistry
Agro-chemicals
Drug develop-ment
Anti-venomics
Medical research
Evolution of venom apparatus
Genome evolution
Gene evolution
Phylogeny of venom proteins
Proteins Peptides
Production systems
Activity screenings
Proteins
Biological structures
Neglected venomous species
Computation
predator-prey co-evolution
Bioassay tests
Transcript-omics
Proteomics
Genomics
Morphology
Venomics
toxin compositions
Scientific strategies
Public outreach
Translational applications
Citizen science
CROSS SUBJECTS

Figure2 Click here to access/download;Figure;Figure2.png

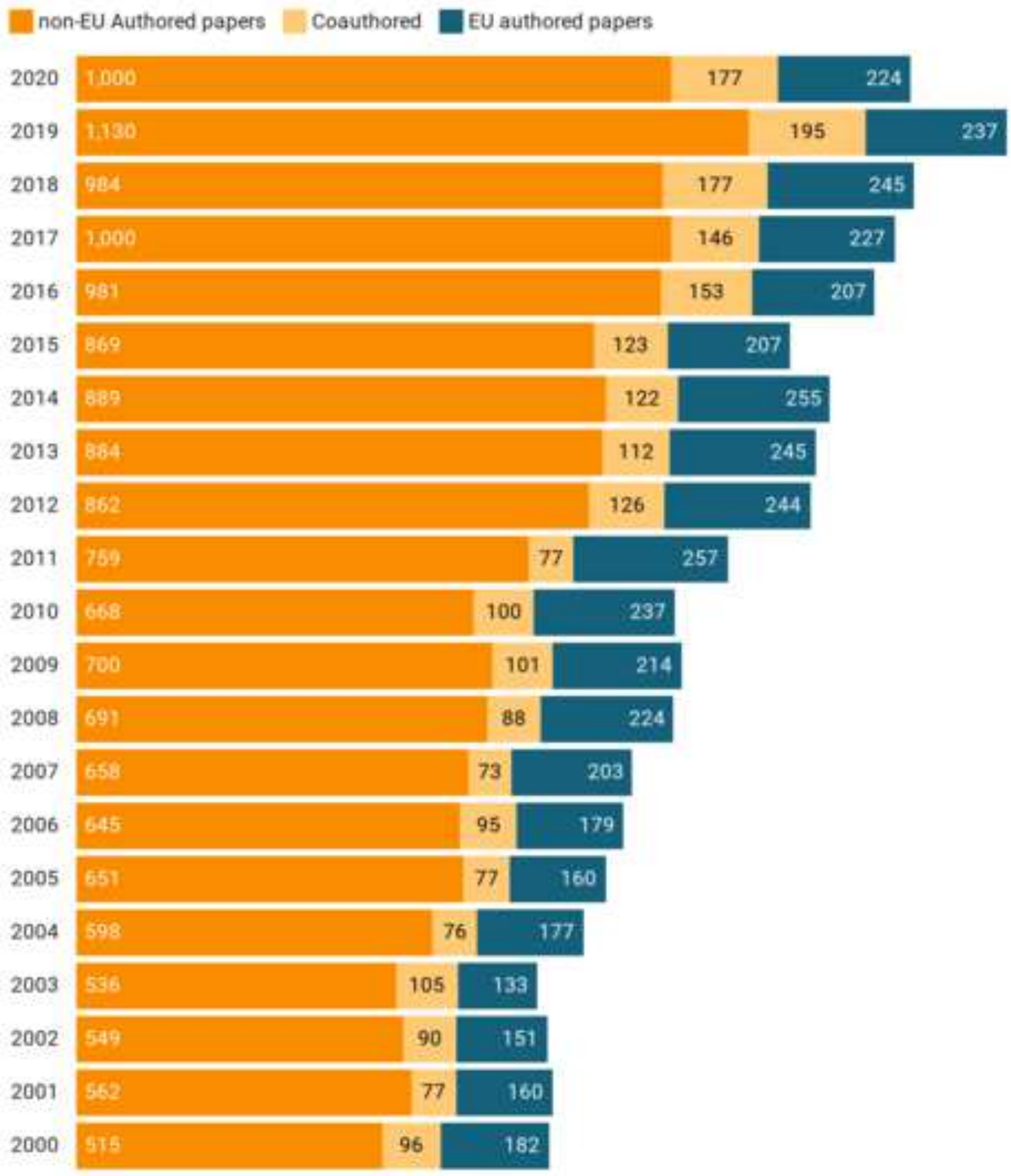

Supplement: giab019_GIGA-D-21-00035_Revision_1 [file giab019_giga-d-21-00035_revision_1.pdf]
