# Supplementary material for: The new COST Action European Venom Network (EUVEN)—synergy and future perspectives of modern venomics
Source: Gigascience. 2021 Mar 25;10(3):giab019. doi: 10.1093/gigascience/giab019 (PMC7992391; doi:10.1093/gigascience/giab019)
Supplement: giab019_Response_to_Reviewer_Comments_Original_Submission [file giab019_response_to_reviewer_comments_original_submission.pdf]

Author's Response To Reviewer Comments

Close

Reviewer #1: This manuscript informs the scientific community, particularly those researchers in the field of molecular toxinology, of the launch of the COST initiative "Action CA19144 - European Venom Network", aimed at promoting synergistic interactions among different stakeholders and foster venom research at the European level. This announcement is clearly formulated and the content and objectives of the COST action discussed. Nothing to criticize in this regard. My only criticism of the content of the manuscript is of Figure 2. I just don't see any point in highlighting a comparison between the EU and extra-EU authors contributions to venom research just referring to the number of indexed publications on venom between the years 2000 and 2020. Number of publications does not tell anything about the global quality of the research! As an example of a serious and informative bibliometric analysis, I would recommend authors to read Groneberg DA, Geier V, KlingelhoÈfer D,Gerber A, Kuch U, Kloft B (2016) Snakebite Envenoming- A Combined Density Equalizing Mapping and Scientometric Analysis of the Publication History. PLoS Negl Trop Dis 10(11): e0005046. doi:10.1371/journal.pntd.0005046.

Authors' response:
The application of bibliometric indicators for assessing scientific performance has always been a controversial issue, and indeed the exhaustive approach used by Groneberg et al. for evaluating publications on a subtopic of venom research is an important step forward. However, such a comprehensive scientometric analysis is beyond the scope of our commentary. The number of publications in peer-reviewed journals over a certain time period is a common and intuitive way to broadly assess the involvement of the research community in a given topic, which is exactly our aim in this commentary. We would instead refrain here from evaluating the quality of European venom research as a whole, since its complexity (due to its recognized multidisciplinarity) would require extensive effort and better fit a separate publication. However, the lack of a qualitative scientometric evaluation of venom research output suggests that such a broad-scale effort may indeed constitute an additional goal of the Action itself, and we will take this possibility in the due consideration along its implementation.

It would also be desirable to refer to web page links where an interested reader could find all the documentation on this Cost Action, including information on the participants and the detailed objectives of the project.

Authors response:
The reference to the Action website, where all relevant information can be easily found, is provided at the beginning of the paragraph The COST Action EUVEN: from fragmentation to integration

****
Reviewer #2: The commentary article The new COST Action European Venom Network (EUVEN) - synergy and future perspectives of modern venomics describes the multidisciplinary nature of venom research and presents the EUVEN, a new EU COST action, as a means of uniting stakeholders in distinct fields. The manuscript presents a brief summary of recent and on-going developments in venom research, highlighting the multi-faceted nature of modern venom research. The authors specifically note the utility of high-throughput molecular characterization and quantification methods (e.g. RNAseq, qMS), the integration of bioinformatic techniques for data processing, and biophysical and in silico approaches for studying molecular characteristics and modes of toxin action. The authors then introduce EUVEN as a research network with the aim of strengthening collaboration and generating synergy among traditionally disparate venom research groups in the EU. Working as a collective, EUVEN will establish best practices in venom research as well as identify and integrate emerging technologies. EUVEN will operate through five working groups focused on target outcomes such as building interaction with industry and multidisciplinary training. The article concludes that EUVEN will provide the resources to develop synergy among participants and stakeholders for transformative work in venom research.
I found the article well written and well-organized. The background provided is appropriate and describes the multidisciplinary nature of the field which presently challenges venom research groups that have traditionally focused on specific research areas. The second half of the article describes how EUVEN

will address these challenges. Most of my comments are very minor and provide some suggestions about grammar and wording. I would encourage the authors to revisit Figure 1 somewhat.
Figure 1 is a very nice-looking figure and in the broad sense fairly intuitive -- when I look at the figure it conveys that venom research is multifaceted and involves many different fields/subfields. However, when I started trying to interpret different parts of the figure things became less clear to me. Specifically, it is not entirely apparent to me what the connections are between different parts of the figure. Do all arrows indicate 'drivers', such that interior arrows are methodological drivers and exterior arrows external drivers? Similarly, how would the authors classify the white ring, grey ring, and grey circles in the main circle? Fields and subfields? Finally, it is not clear to me exactly what the roles of the grey rectangles at the top of the bottom of the figure are. I realize these fields indicate the working groups and cross subjects, but I am not sure how they link to the rest of the figure as there does not seem to be any correspondence with main parts of
the circle—at present it seems like they could be removed with no effect on the message the figure conveys. I believe having a little more information on how this figure is organized would greatly enrich its interpretation and utility. I believe most of the relevant information can be provided in the figure caption, though perhaps the authors could convey this information in a figure legend or something if they prefer.
I have included my other minor comments below and on the included pdf (comments are the same here and on the pdf). I commend the authors on a well-constructed manuscript, and I look forward to both seeing the manuscript in press and the future work and outcomes of EUVEN more broadly.

Authors' response:
We have modified the figure caption as follows in order to convey the required information, explicitly describing the conceptual links between the different parts of the figure.
"The multidisciplinary, integrative and interconnected vision of venom research proposed in EUVEN. The centre is composed of modern morphology and -omics methods, in particular proteomics, transcriptomics and genomics. In the surrounding white circle, main aspects of current venom research are indicated and summarized by the major topics in the oval grey circles. These major topics are loosely associated with broader themes given in the grey circle. The whole system is affected and interacts with outer drivers (in purple). This integrative scheme is the heart of EUVEN, in which five major Working Groups focus on these topics and methods (top grey bar; see text for details). The cross subjects in which the scientific, technological and socio-economic impact of EUVEN will be realized are outlined in the lower grey bar."

-Andrew Mason

Minor comments:

Page 2, line 9 (under Background), First sentence of paragraph 2: I just wanted to verify that Barua et al. 2019 is citation that the authors intended to use here.
My reading of Barua et al. 2019 is that it does not provide direct evidence of venoms being fast acting at low concentrations or being physiologically specific, but rather presents evidence of adaptive convergence in snakes. To me, this citation would be better suited to supporting the previous sentence ('Venom toxins are adaptive and highly convergent traits...') while something like the Casewell et al. 2013 'Complex cocktails' review, which includes a summary of some evidence for prey specificity in venom, would better support this sentence.
Authors' response:
The reference suggested by the reviewer is indeed more appropriate. We have replaced Barua et al. with Casewell et al.

Page 2, line 11 (under Background), "Ten animal derived drugs have been so far approved": The placement of 'so far' reads just a bit awkwardly to me, though I do not think it is grammatically incorrect. I would write the sentence as 'Ten animal-derived drugs so far have been approved' or even 'To date, ten animal-derived drugs have been approved and ...'
Authors' response:
We have modified the text according to the reviewer's suggestion.

Page 4, line 14, "The European Cooperation in Science and Technology (COST) Association operates": change 'operates' to 'has operated'
Authors' response:
We have modified the text according to the reviewer's suggestion.

Page 4, line 23, "EUVEN aims at involving also": change to "EUVEN also aims to involve"
Authors' response:
We have modified the text according to the reviewer's suggestion.

Page 4, line 24: remove comma after "biodiversity-based research"
Authors' response:
We have modified the text according to the reviewer's suggestion.

Page 4, line 31: remove comma after "comparable results"
Authors' response:
We have modified the text according to the reviewer's suggestion.

Page 5, line 4, "transform discoveries and knowledge of researchers into": I would recommend changing "knowledge of researchers" to either "research knowledge" or "researchers' knowledge" depending on what the authors would like to convey.
Authors' response:
We have modified the text according to the reviewer's suggestion.

Page 5, line 14, first sentence under Conclusion, to overcome the lack of coordination, tools, and resources, and develop a fully synergistic network.: think there is something a bit off about the wording here. It seems like 'develop a fully synergistic network' should either be presented as an outcome (as in "to overcome the lack of coordination, tools, and resources to develop a fully synergistic network") or as a means of accomplishment (as in "to overcome the lack of coordination, tools, and resources through the development of a fully synergistic network"). But that could just be how I was expecting the sentence to be structured and perhaps as it is written is exactly what the authors wanted to convey.
Authors' response:
We have modified the text according to the reviewer's suggestion.

Page 5, line 20, "building an effective network, able to bridge": an effective network that is able to bridge
Authors' response:
We have modified the text according to the reviewer's suggestion.

Page 5, line 21: remove comma after sector
Authors' response:
We have modified the text according to the reviewer's suggestion.

Page 5, line 21, extraordinary: should be extraordinarily
Authors' response:
We have modified the text according to the reviewer's suggestion.

Figure 2 legend, to obtain publications lists: to obtain publication lists
Authors' response:
We have modified the text according to the reviewer's suggestion.

References, 10: I don't believe that there was a 10th citation in the text.
Authors' response:
We have removed this reference.

Close
