# Supplementary material for: The new COST Action European Venom Network (EUVEN)—synergy and future perspectives of modern venomics
Source: Gigascience. 2021 Mar 25;10(3):giab019. doi: 10.1093/gigascience/giab019 (PMC7992391; doi:10.1093/gigascience/giab019)
Supplement: giab019_Reviewer_1_Report_Original_Submission — Juan Calvete -- 2/10/2021 Reviewed [file giab019_reviewer_1_report_original_submission.pdf]

Reviewer Report

Title: The new COST Action European Venom Network (EUVEN) â€“ synergy and future perspectives of modern venomics

Version: Original Submission **Date:** 2/10/2021

Reviewer name: Juan Calvete

Reviewer Comments to Author:

This manuscript informs the scientific community, particularly those researchers in the field of molecular toxinology, of the launch of the COST initiative "Action CA19144 - European Venom Network", aimed at promoting synergistic interactions among different stakeholders and foster venom research at the European level. This announcement is clearly formulated and the content and objectives of the COST action discussed. Nothing to criticize in this regard. My only criticism of the content of the manuscript is of Figure 2. I just don't see any point in highlighting a comparison between the EU and extra-EU authors contributions to venom research just referring to the number of indexed publications on venom between the years 2000 and 2020. Number of publications does not tell anything about the global quality of the research! As an example of a serious and informative bibliometric analysis, I would recommend authors to read Groneberg DA, Geier V, KlingelhoÃˆfer D, Gerber A, Kuch U, Kloft B (2016) Snakebite Envenoming- A Combined Density Equalizing Mapping and Scientometric Analysis of the Publication History. PLoS Negl Trop Dis 10(11): e0005046. doi:10.1371/journal.pntd.0005046.
It would also be desirable to refer to web page links where an interested reader could find all the documentation on this Cost Action, including information on the participants and the detailed objectives of the project.

Level of Interest

Please indicate how interesting you found the manuscript: Choose an item.

Quality of Written English

Please indicate the quality of language in the manuscript: Choose an item.

Declaration of Competing Interests

Please complete a declaration of competing interests, considering the following questions:

- Have you in the past five years received reimbursements, fees, funding, or salary from an organisation that may in any way gain or lose financially from the publication of this manuscript, either now or in the future?
- Do you hold any stocks or shares in an organisation that may in any way gain or lose financially from the publication of this manuscript, either now or in the future?

- Do you hold or are you currently applying for any patents relating to the content of the manuscript?
- Have you received reimbursements, fees, funding, or salary from an organization that holds or has applied for patents relating to the content of the manuscript?
- Do you have any other financial competing interests?
- Do you have any non-financial competing interests in relation to this paper?

If you can answer no to all of the above, write 'I declare that I have no competing interests' below. If your reply is yes to any, please give details below.

I declare that I have no competing interests

I agree to the open peer review policy of the journal. I understand that my name will be included on my report to the authors and, if the manuscript is accepted for publication, my named report including any attachments I upload will be posted on the website along with the authors' responses. I agree for my report to be made available under an Open Access Creative Commons CC-BY license (http://creativecommons.org/licenses/by/4.0/). I understand that any comments which I do not wish to be included in my named report can be included as confidential comments to the editors, which will not be published.

Choose an item.

To further support our reviewers, we have joined with Publons, where you can gain additional credit to further highlight your hard work (see: https://publons.com/journal/530/gigascience). On publication of this paper, your review will be automatically added to Publons, you can then choose whether or not to claim your Publons credit. I understand this statement.

Yes Choose an item.
