# Supplementary material for: The new COST Action European Venom Network (EUVEN)—synergy and future perspectives of modern venomics
Source: Gigascience. 2021 Mar 25;10(3):giab019. doi: 10.1093/gigascience/giab019 (PMC7992391; doi:10.1093/gigascience/giab019)
Supplement: giab019_Reviewer_2_Report_Original_Submission — Andrew J Mason -- 2/16/2021 Reviewed [file giab019_reviewer_2_report_original_submission.pdf]

Reviewer Report

Title: The new COST Action European Venom Network (EUVEN) â€“ synergy and future perspectives of modern venomics

Version: Original Submission **Date:** 2/16/2021

Reviewer name: Andrew J Mason

Reviewer Comments to Author:

The commentary article The new COST Action European Venom Network (EUVEN) - synergy and future perspectives of modern venomics describes the multidisciplinary nature of venom research and presents the EUVEN, a new EU COST action, as a means of uniting stakeholders in distinct fields. The manuscript presents a brief summary of recent and on-going developments in venom research, highlighting the multi-faceted nature of modern venom research. The authors specifically note the utility of high-throughput molecular characterization and quantification methods (e.g. RNAseq, qMS), the integration of bioinformatic techniques for data processing, and biophysical and in silico approaches for studying molecular characteristics and modes of toxin action. The authors then introduce EUVEN as a research network with the aim of strengthening collaboration and generating synergy among traditionally disparate venom research groups in the EU. Working as a collective, EUVEN will establish best practices in venom research as well as identify and integrate emerging technologies. EUVEN will operate through five working groups focused on target outcomes such as building interaction with industry and multidisciplinary training. The article concludes that EUVEN will provide the resources to develop synergy among participants and stakeholders for transformative work in venom research.
I found the article well written and well-organized. The background provided is appropriate and describes the multidisciplinary nature of the field which presently challenges venom research groups that have traditionally focused on specific research areas. The second half of the article describes how EUVEN will address these challenges. Most of my comments are very minor and provide some suggestions about grammar and wording. I would encourage the authors to revisit Figure 1 somewhat. Figure 1 is a very nice-looking figure and in the broad sense fairly intuitive -- when I look at the figure it conveys that venom research is multifaceted and involves many different fields/subfields. However, when I started trying to interpret different parts of the figure things became less clear to me. Specifically, it is not entirely apparent to me what the connections are between different parts of the figure. Do all arrows indicate 'drivers', such that interior arrows are methodological drivers and exterior arrows external drivers? Similarly, how would the authors classify the white ring, grey ring, and grey circles in the main circle? Fields and subfields? Finally, it is not clear to me exactly what the roles of the grey rectangles at the top of the bottom of the figure are. I realize these fields indicate the working groups and cross subjects, but I am not sure how they link to the rest of the figure as there does not seem to be any correspondence with main parts of the circleâ€”at present it seems like they could be removed with no effect on the message the figure conveys. I believe having a little more information on how this figure is organized would greatly enrich its interpretation and utility. I believe most of the relevant information can be provided in the figure caption, though perhaps the authors could convey

this information in a figure legend or something if they prefer.
I have included my other minor comments below and on the included pdf (comments are the same here and on the pdf). I commend the authors on a well-constructed manuscript, and I look forward to both seeing the manuscript in press and the future work and outcomes of EUVEN more broadly.
-Andrew Mason
Minor comments:
Page 2, line 9 (under Background), First sentence of paragraph 2: I just wanted to verify that Barua et al. 2019 is citation that the authors intended to use here.
My reading of Barua et al. 2019 is that it does not provide direct evidence of venoms being fast acting at low concentrations or being physiologically specific, but rather presents evidence of adaptive convergence in snakes. To me, this citation would be better suited to supporting the previous sentence ('Venom toxins are adaptive and highly convergent traits...') while something like the Casewell et al. 2013 'Complex cocktails' review, which includes a summary of some
evidence for prey specificity in venom, would better support this sentence.
Page 2, line 11 (under Background), "Ten animal derived drugs have been so far approved": The placement of 'so far' reads just a bit awkwardly to me, though I do not think it is grammatically incorrect.
I would write the sentence as 'Ten animal-derived drugs so far have been approved' or even 'To date, ten animal-derived drugs have been approved and ...'
Page 4, line 14, "The European Cooperation
in Science and Technology (COST) Association operates": change 'operates' to 'has operated'
Page 4, line 23, "EUVEN aims at involving also": change to "EUVEN also aims to involve"
Page 4, line 24: remove comma after "biodiversity-based research"
Page 4, line 31: remove comma after "comparable results"
Page 5, line 4, "transform discoveries and knowledge of researchers into": I would recommend changing "knowledge of researchers" to either "research knowledge" or "researchers' knowledge" depending on what the authors would like to convey.
Page 5, line 14, first sentence under Conclusion, to overcome the lack of
coordination, tools, and resources, and develop a fully synergistic network.: think there is something a bit off about the wording here. It seems like 'develop a fully synergistic network' should either be presented as an outcome (as in "to overcome the lack of coordination, tools, and resources to develop a fully synergistic network") or as a means of accomplishment (as in "to overcome the lack of coordination, tools, and resources through the development of a fully synergistic network"). But that could just be how I was expecting the sentence to be structured and perhaps as it is written is exactly what the authors wanted to convey.
Page 5, line 20, "building an effective network, able to bridge": an effective network that is able to bridge
Page 5, line 21: remove comma after sector
Page 5, line 21, extraordinary: should be extraordinarily
Figure 2 legend, to obtain publications lists: to obtain publication lists
References, 10: I don't believe that there was a 10th citation in the text.

Level of Interest

Please indicate how interesting you found the manuscript: Choose an item.

Quality of Written English

Please indicate the quality of language in the manuscript: Choose an item.

Declaration of Competing Interests

Please complete a declaration of competing interests, considering the following questions:

- Have you in the past five years received reimbursements, fees, funding, or salary from an organisation that may in any way gain or lose financially from the publication of this manuscript, either now or in the future?
- Do you hold any stocks or shares in an organisation that may in any way gain or lose financially from the publication of this manuscript, either now or in the future?
- Do you hold or are you currently applying for any patents relating to the content of the manuscript?
- Have you received reimbursements, fees, funding, or salary from an organization that holds or has applied for patents relating to the content of the manuscript?
- Do you have any other financial competing interests?
- Do you have any non-financial competing interests in relation to this paper?

If you can answer no to all of the above, write 'I declare that I have no competing interests' below. If your reply is yes to any, please give details below.

I declare that I have no competing interests

I agree to the open peer review policy of the journal. I understand that my name will be included on my report to the authors and, if the manuscript is accepted for publication, my named report including any attachments I upload will be posted on the website along with the authors' responses. I agree for my report to be made available under an Open Access Creative Commons CC-BY license (http://creativecommons.org/licenses/by/4.0/). I understand that any comments which I do not wish to be included in my named report can be included as confidential comments to the editors, which will not be published.

Choose an item.

To further support our reviewers, we have joined with Publons, where you can gain additional credit to further highlight your hard work (see: https://publons.com/journal/530/gigascience). On publication of

this paper, your review will be automatically added to Publons, you can then choose whether or not to claim your Publons credit. I understand this statement.

Yes Choose an item.
